# Investigation on the Efficiency of Chinese Herbal Injections for Treating Non-small Cell Lung Cancer With Vinorelbine and Cisplatin Based on Multidimensional Bayesian Network Meta-Analysis

**DOI:** 10.3389/fphar.2020.631170

**Published:** 2021-01-29

**Authors:** Mengwei Ni, Haojia Wang, Miaomiao Wang, Wei Zhou, Jingyuan Zhang, Jiarui Wu, Dan Zhang, Zhiwei Jing, Xinkui Liu, Zhishan Wu, Siyu Guo, Shanshan Jia, Xiaomeng Zhang, Xiaoguang Sheng

**Affiliations:** ^1^ Department of Clinical Chinese Pharmacy, School of Chinese Materia Medica, Beijing University of Chinese Medicine, Beijing, China; ^2^ China Academy of Chinese Medicine Sciences, Beijing, China

**Keywords:** Chinese herbal injection, vinorelbine plus cisplatin, non-small cell lung cancer, network meta-analysis, systematic review, multidimensional cluster analysis

## Abstract

**Background:** As non-small cell lung cancer (NSCLC) seriously threatens human health, several clinical studies have reported that Chinese herbal injections (CHIs) combined with vinorelbine and cisplatin (NP) are beneficial. This multidimensional network meta-analysis was performed to explore the preferable options among different CHIs for treating NSCLC.

**Methods:** A literature search was performed in several databases to identify randomized controlled trials (RCTs) of CHIs in the treatment of NSCLC from inception to January 31, 2019. Final included studies met the eligibility criteria and methodological quality recommendations. Data analysis was performed using Stata 13.0 and WinBUGS 14.0 software. Each outcome was presented as an odds ratio and the surface under the cumulative ranking curve value (SCURA). The “scatterplot3d” package in R 3.6.1 software was used to perform multidimensional cluster analysis.

**Results:** Ultimately, 97 eligible RCTs involving 7,440 patients and 14 CHIs were included in this network meta-analysis. Combined with NP chemotherapy, Kanglaite injection plus NP exhibited a better impact on the clinical effectiveness rate (SCURA probability: 78.34%), and Javanica oil emulsion injection plus NP was better in the performance status (95.44%). Huachansu injection plus NP was dominant in reducing thrombocytopenia (92.67%) and gastrointestinal reactions (92.52%). As to multidimensional cluster analysis, Shenmai injection plus NP was superior considering improving the clinical effectiveness rate, performance status and relieving leukopenia.

**Conclusions:** The combination of CHIs and NP has a better impact on patients with NSCLC than NP alone. Among them, Shenmai injection plus NP, Kanglaite injection plus NP and Javanica oil emulsion injection plus NP were notable. Nevertheless, more multicenter and better designed RCTs are needed to validate our findings.

## Introduction

The global cancer statistics suggests that the number of new cancer cases and cancer deaths in 2018 are 18.1 million and 9.6 million. Lung cancer, the most commonly diagnosed cancer (11.6%) and the leading cause of cancer death (18.4%), has a poor prognosis, with a five-year survival of only 16.8% (Ferlay et al., 2015; Bray et al., 2018; Ettinger et al., 2013). Based on histology, lung cancer is separated into non-small cell lung cancer (NSCLC) and small cell lung cancer (SCLC) (Siegel et al., 2018). Approximately 85% of lung cancer patients are NSCLC, and in which 50% present at advanced stages at the time of their first diagnosis (La Fleur et al., 2019). With treatments for NSCLC improving in recent years, platinum-based two-drug combination chemotherapy regimens have become the primary therapeutic regimens, including vinorelbine plus cisplatin (NP), paclitaxel plus cisplatin, and gemcitabine plus cisplatin (Maione et al., 2011). NP is one of the most commonly used chemotherapy drugs for the clinical treatment of NSCLC, and its antineoplastic efficacy has been endorsed (Rinaldi et al., 2006; Li et al., 2014). However, this treatment is often accompanied by a highly toxic physiological environment and adverse events (Pfister et al., 2004).

In China, the method of using a combination of traditional Chinese medicine (TCM) and chemotherapy has been widely adopted in the treatment of cancer (Qi et al., 2010). The beneficial role of TCM in adjuvant treatment of cancer has been reported in many studies, specifically in retarding cancer progression and ameliorating chemotherapy-induced complications and adverse events (Hofseth and Wargovich, 2007; Konkimalla and Efferth, 2008). Chinese herbal injections (CHIs), an indispensable part of TCM, is considered more and more important in treating cancer. Many studies have shown that the active ingredients of the Aidi injection, such as cantharidin, ginsenosides, and astragalosides, have antitumor and immunomodulatory effects in lung cancer (Huang et al., 2011; Zhang et al., 2014; Cichello et al., 2015; Xiao et al., 2016; Ge et al., 2017; Hsieh et al., 2017; Meng et al., 2018). The active ingredients in ginseng can increase the accumulation of apoptotic proteins in the mitochondrial and downregulate the expression of antiapoptotic proteins. Studies have shown that ginseng can activate macrophages and NK cells, which may be related to its antitumor effect (Yoon et al., 2004; Hsia et al., 2016; Dai et al., 2017; Jiang et al., 2017; Wang J et al., 2018a; Wang JJ et al., 2018b; Majeed et al., 2018).

**FIGURE 1:**
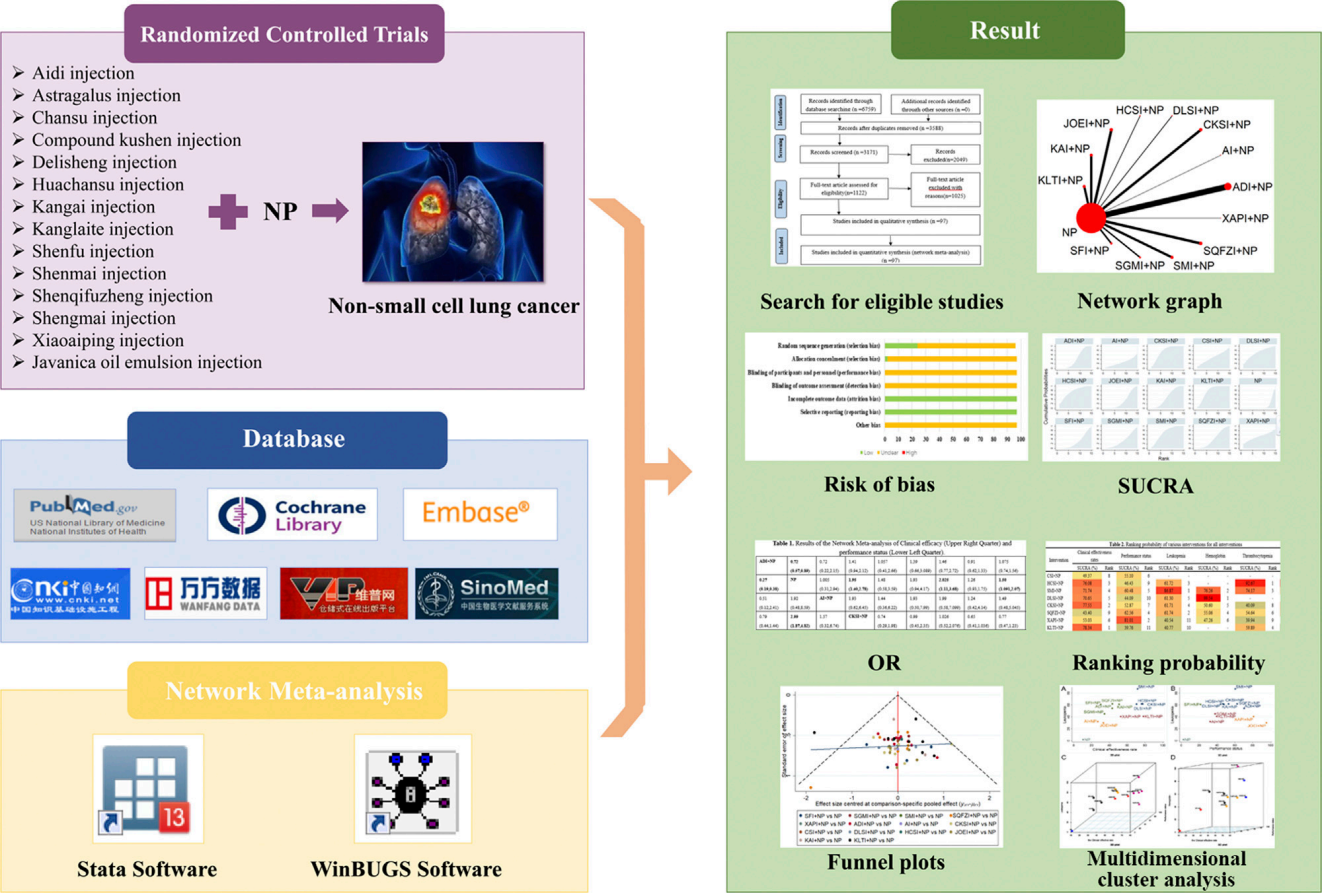
Graphical abstract of the network meta-analysis. Note: NP, vinorelbine plus cisplatin; SUCRA, surface under the cumulative ranking curve; OR, odds ratio.

However, there are various types of CHIs, and the optimal strategy for combining CHIs with chemotherapy for treating NSCLC remains inconclusive, which may cause difficulty for clinicians in clinical treatment. Network meta-analyses (NMAs) can integrate the available comparisons based on clinical trials and simultaneous various interventions to assess their comparative efficacy (Salanti et al., 2014). Recently, NMA has increasingly been considered as a vital methodology by health care decision makers and clinical researchers (Ioannidis, 2006; Edwards et al., 2009; Piccini and Kong, 2011; Laws et al., 2019). This NMA compared the efficacy of combining 14 CHIs with NP for treating NSCLC by quantitatively synthesizing the evidence. The objective of this NMA was to supplement the optimal strategy of NSCLC treatment and to strengthen additional insights for clinical practice in the future. The graphical abstract of this NMA is presented in Figure 1.

## Methods

The procedure of the current NMA was performed in accordance with the Preferred Reporting Items for Systematic Reviews and Meta-Analyses (PRISMA) Extension Statement for Reporting of Systematic Reviews Incorporating Network Meta-analyses of Health Care Interventions (Hutton et al., 2015). A completed PRISMA check list is included as Supplementary material Presentation File 1.

### Search Strategy

In this NMA, a comprehensive literature search was performed using electronic databases including embase, PubMed, the Cochrane Library, the China National Knowledge Infrastructure database, the Wanfang database, the CQVIP database and the China Biology Medicinedisc without restrictions on the publication year, language, or blinding methods. The retrieval period was from inception to January 31, 2019. To identify relevant publications, the method of combining MeSH terms with free text search terms was applied to the search, which focused on three themes: 1) NSCLC, 2) CHIs, and 3) study type (randomized controlled trials (RCTs)). Using PubMed as an example, the following terms were used for NSCLC: “Non-Small-Cell Lung Carcinomas [MeSH Terms],” “Non-Small-Cell Lung Carcinoma,” “Nonsmall Cell Lung Cancer,” “Non Small Cell Lung Carcinoma,” “Non-Small Cell Lung Carcinoma,” and “Non-Small Cell Lung Cancer.” Details on the retrieval strategies are provided in Supplementary material Presentation File 1.

### Inclusion Criteria

#### Types of Studies

The study was an RCT that compared the relative outcomes of CHIs combined with NP for treating NSCLC. No limitations on language, publication year, or publication status was applied. Only the first publication will be included if there are duplicate studies. Studies were excluded if the study designs and publication types were duplicates, and the full text was unavailable.

#### Types of Participants

The study included patients with NSCLC in stage Ⅲ or Ⅳ and were diagnosed by cytology or pathology without limitations on sex, age, race, region or nationality. Patients with NSCLC who had other tumors were excluded.

#### Types of Interventions

The study conducted a comparison with NSCLC patients receiving NP alone or in combination with another CHI, disregarding its course or dosage. Interventions included the combined application of CHIs and NP in either arm of treatment, and the CHIs used (such as Shenfu injection, Shenmai injection, and Shenqifuzheng injection) were applied in clinics for treating tumors and authorized by the China Food and Drug Administration. If patients had complications during the treatment, then some appropriate mitigation measures could be taken. The interventions included surgery, radiotherapy or other cancer treatments were excluded.

#### Types of Outcomes

The study described efficacy outcomes including the clinical effectiveness rate and the performance status, and adverse drug reactions and adverse drug events (ADRs/ADEs), such as leukopenia, hemoglobin reduction, thrombocytopenia and gastrointestinal reactions (Sylvester, 1980; People's Republic of China Department of Health Management, 1991). 1) Clinical effectiveness rate. According to the WHO criteria for evaluating the efficacy of solid tumors, the clinical effectiveness rate can be divided into four levels: complete response (CR), which means that patients’ visible lesions disappeared completely >1 month after the end of treatment; partial response (PR) means that the tumor area of a single lesion was reduced by ≥ 50%, or the sum of the vertical diameter products of the two largest tumors in multiple lesions was reduced by >50%; stable disease (SD), with no significant change within at least 4 weeks, and estimated tumor size increased by <25% or decreased by <50%; and progressive disease (PD), which means that the estimated size of the new or original lesion had increased by ≥ 25%. The clinical effectiveness rate of this study was calculated by the following formula: the clinical effectiveness rate = (CR + PR)/total number of patients × 100%. 2) Performance status. The performance status was evaluated by Karnofsky performance tstatus (KPS) score. The improvement of KPS score by ≥ 10 points after treatment was considered to improve the motor state; a decrease of ≥10 points in KPS score was considered to reduce the performance status; while the increase or decrease of KPS score <10 points was considered to be stable. Performance improvement rate = number of patients with improved performance/total number of patients × 100%. 3) Leukopenia, hemoglobin reduction, thrombocytopenia and gastrointestinal reactions. They were evaluated according to the “Acute and Subacute Toxicity Standards of Chemotherapy Drugs” formulated by the WHO in 1981. The ADRs/ADEs were divided into 5 grades. The incidence of ADR/ADEs = number of patients with ADRs/total number of patients × 100%.

### Data Extraction and Quality Assessment

All citations were managed and organized via NoteExpress software. After duplicate records being removed, the two investigators independently screened the titles and abstracts of the articles. Further assessment of the potential articles was based on full-text versions. Any discrepancies of opinions were resolved by discussion or consultating a third reviewer. The following data were recorded in a form predesigned. 1) Publication information: the first author name and the publication year. 2) Number, age, sex and other characteristics of the enrolled patients with NSCLC, as well as the cancer type and stage. 3) Dosage, duration, treatment cycle and other information of interventions. 4) The measured data on the efficacy outcomes. 5) Important items for quality evaluation, such as blinding, randomized allocation methods, and so on.

The quality of eligible RCTs were evaluate using the Cochrane risk of bias tool (Cochrane Handbook for Systematic Reviews of Interventions, version 5.1.0) (Higgins and Green, 2011). The quality assessment was performed by two reviewers, and any discrepancies during this process were solved by discussion or through adjudication by a third investigator. The following domains were assessed: selection bias (random sequence generation and allocation concealment), performance bias (blinding of the participants and personnel), detection bias (blinding of the outcome assessment), attrition bias (incomplete outcome data), reporting bias (selective reporting) and other bias. Each bias has three levels: “low risk”, “unclear risk” and “high risk”.

### Data Analysis

For each outcome, we carried out a Bayesian NMA to compare the efficacy among the eligible CHIs combined with NP for treating NSCLC. WinBUGS 14.0 software (MRC Biostatistics Unit, Cambridge, UK) was used to perform statistical analysis. The comparative efficacies of the treatments are expressed as odds ratios (ORs) with 95% confidence intervals (95% CIs) for dichotomous outcomes. The differences between the compared groups were deemed significant when the 95% CI of the OR did not contain 1.00. The results of the analysis procedure were based on 200,000 simulation iterations, and 10,000 adaptation iterations were used for the annealing algorithm to eliminate the impact of the initial value. Additionally, all graphs of the NMA were presented using Stata 13.1 software (Stata Corp, College Station, TX) (Shim et al., 2017). The network graph illustrates the relationships of interventions. The width of the line in the network graph is proportional to the number of RCTs included in this comparison, and the node size corresponds to the total sample size of this intervention (Chaimani et al., 2013; Donegan et al., 2013). The surface under the cumulative ranking curve (SUCRA) was used to estimate the ranking probabilities for different CHIs, which ranged from 0 to 100%. A better treatment was indicated by a higher SUCRA (Rücker and Schwarzer, 2015; Trinquart et al., 2016; Chang and Guo, 2017; Cai et al., 2018). A comparison-adjusted funnel plot was also constructed to graphically estimate the publication bias. If the comparison-adjusted funnel plot was symmetrical, there was no obvious publication bias (Trinquart et al., 2012; Krahn et al., 2014). A cluster analysis was also performed to determine the most efficacious injection in the treatment of NSCLC. Interventions located in the upper-right corner were superior to others (Veroniki et al., 2015).

In order to detect the amount and source of heterogeneity among included RCTs, we used the Review Manager 5.3 software (The Nordic Cochrane Center, Copenhagen, Denmark) to conduct a meta-analysis of the results of direct comparison between CHI + NP and NP analysis for each outcome. The heterogeneity within each injection subgroup was analyzed by Cochrane's *Q* test, and the *p*-value was used to evaluate the degree of heterogeneity. When *p* > 0.05, the difference within a group is considered small and the heterogeneity is not obvious, while *p* < 0.05 is considered that there is obvious heterogeneity among studies. For outcomes with obvious heterogeneity, covariates that may have an impact on heterogeneity were selected, and were analyzed in meta-regression. Use Stata 13.1 software to implement meta-regression, and specify the restricted maximum likelihood (REML) method to estimate the variance component τ_2_ between studies. Finally, each covariate is introduced into the regression model to determine its relationship with heterogeneity.

The present NMA did not require ethical approval because data was gathered from previously published RCTs.

### Multidimensional Cluster Analysis

In this study, the “scatterplot3d” package in R 3.6.1 software (Mathsoft, Cambridge, United States) was used to perform multidimensional cluster analysis of the outcomes. The k-means method was used to cluster these interventions, and the number of clusters was adjusted according to the actual problem. First, all interventions were randomly divided into k initial classes, and the average data of these k classes were used as initial aggregation points. Next, one intervention was classified as the category of the aggregation point closest to it, and the aggregation point of this category updated to the average of the current outcome indicators. Then re-categorize and classify all interventions. Repeat the above steps until all interventions have been assigned. Finally, a three-dimensional stereo Gram that can visually display clustering results of three outcomes is produced. Different categories of interventions were marked with different colors.

## Results

### Search Results

**FIGURE 2:**
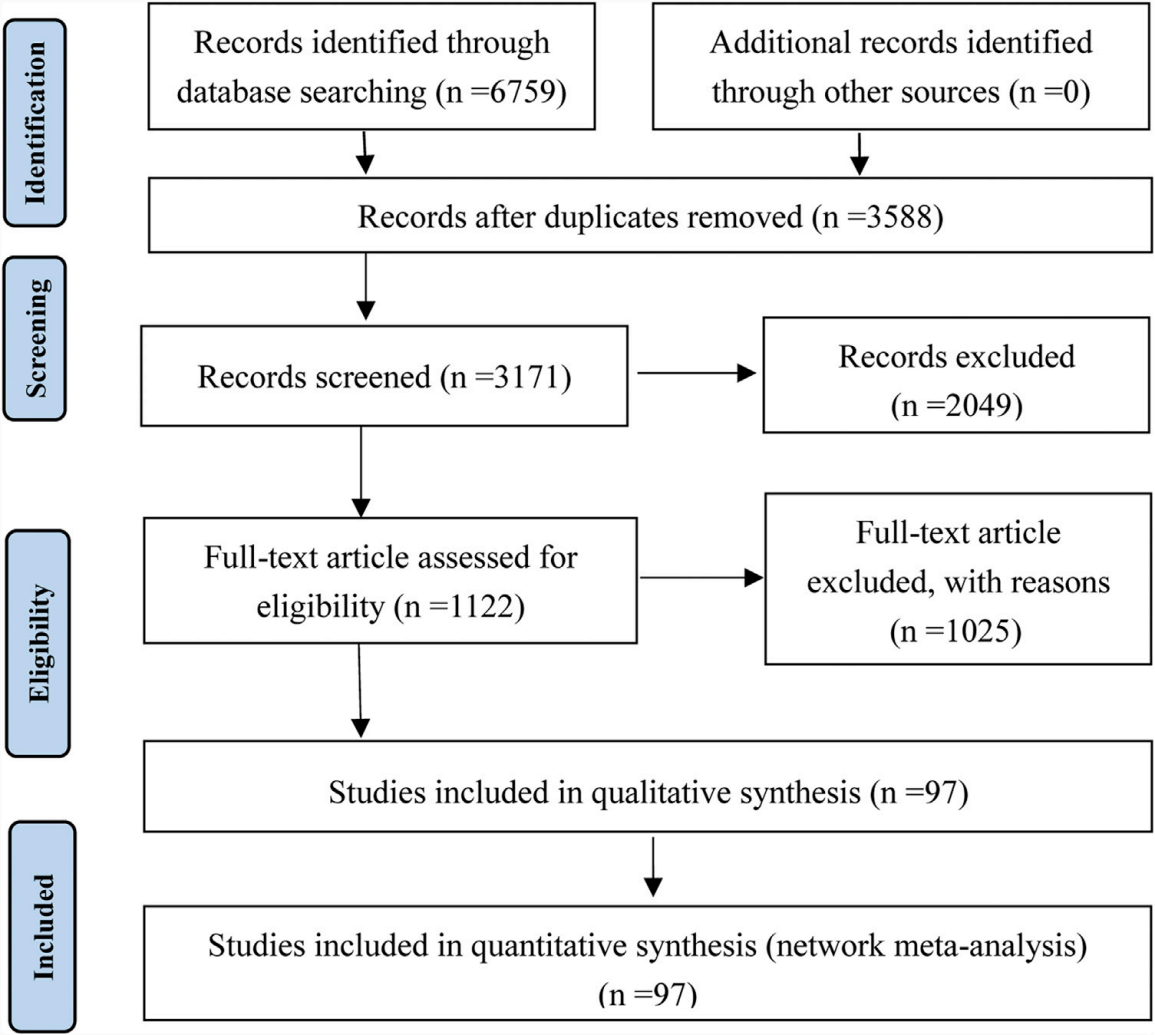
Flow chart of the search for eligible studies.

Initially, the search strategy yielded 6,759 prospective articles from the electronic databases. After excluding 3,588 duplicates and screening the titles and abstracts, 1,122 articles remained for further evaluation. After detailed examination, a total of 97 RCTs involving 14 CHIs met our selection criteria. Information about the included injections were shown in Supplementary material Presentation File 1. The study identification, screening, and inclusion process is illustrated in Figure 2. The number of studies included for different CHIs was as follows: Aidi injection (20 RCTs), *Astragalus* injection (1 RCT), Chansu injection (1 RCT), Compound kushen injection (8 RCTs), Delisheng injection (2 RCTs), Huachansu injection (3 RCTs), Kangai injection (7 RCTs), Kanglaite injection (12 RCTs), Shenfu injection (9 RCTs), Shenma injection (9 RCTs), Shenqifuzheng injection (11 RCTs), Shengmai injection (5 RCTs), Xiaoaiping injection (1 RCTs), and Javanica oil emulsion injection (8 RCTs).

### Inclusion Studies and Characteristics

**FIGURE 3:**
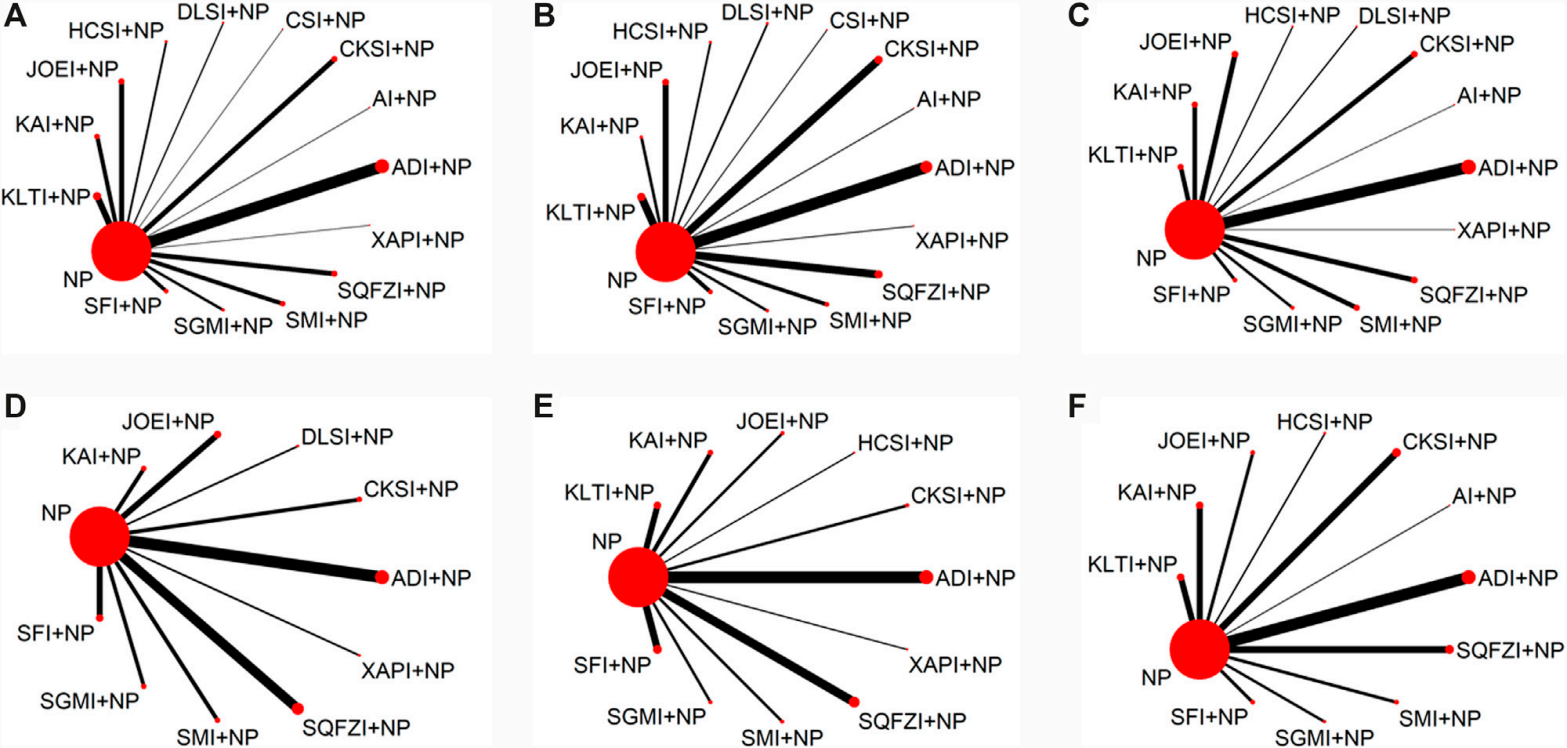
Network graph of outcomes. **(A)** Clinical effectiveness rate; **(B)** Performance status; **(C)** Leukopenia; **(D)** Hemoglobin reduction; **(E)** Thrombocytopenia; **(F)** Gastrointestinal reactions. Note: The width of the lines in the network graph is proportional to the number of RCTs used for the comparisons, and the node sizes correspond to the total sample sizes for the treatments. NP, vinorelbine and cisplatin; ADI, Aidi injection; AI, *Astragalus* injection; CSI, Chansu injection; CKSI, Compound kushen injection; DLSI, Delisheng injection; HCSI, Huachansu injection; JOEI, Javanica oil emulsion injection; KAI, Kangai injection; KLTI, Kanglaite injection; SFI, Shenfu injection; SMI, Shenmai injection; SQFZI, Shenqifuzheng injection; SGMI, Shengmai injection; XAPI, Xiaoaiping injection.

The baseline characteristics of each included RCT are summarized in Table 1. Overall, the 97 RCTs enrolled 7,440 patients with NSCLC; 3,747 of them received a combination of CHIs and NP in the experimental group, and 3,693 patients received only NP in the control group. All RCTs reported the sample size and the patients’ age, sex, tumor node metastasis (TNM) stage, expected survival time, and Karnofsky performance status (KPS) score before treatment. Figure 3 presents the network plot of the interventions included in the Bayesian analysis.

**TABLE 1:** Characteristics of the included randomized controlled trials.

Study ID	Sex (M/F)	N (E/C)	AVG age	KPS score	TNM stages	Pathological type	Therapy of experiment	Therapy of control	Treatment(days)	Outcomes
Han (2012)	46/30	40/36	31–69/32–71	>90	Ⅲb, Ⅳ	NR	ADI 100 ml + NVB 25 mg/m2 + DDP 30 mg/m2	NVB 25 mg/m2 + DDP 30 mg/m2	(10/21)×(2)	①③
Gao (2008)	42/30	36/36	49–68/32–76	NR	Ⅲb, Ⅳ	Lac, LSCC, O	ADI 50 ml + NVB 25 mg/m2 + DDP 50 mg/m2	NVB 25 mg/m2 + DDP 50 mg/m2	(10/21)×(3)	①③
Liu and Wang (2009)	58/22	43/37	49.4 ± 3.3/48.3 ± 3.4*	NR	Ⅲb, Ⅳ	Lac, LSCC, LCLC	ADI 50 ml + NVB 25mg/m2 + DDP 30mg/m2	NVB 25 mg/m2 + DDP 30 mg/m2	42/(21 × 2)	①②③
Xu et al. (2013)	53/17	35/35	35–75/33–76	≥70	Ⅲb, Ⅳ	Lac, LSCC, LASC	ADI 100 ml + NVB 25 mg/m2 + DDP 80 mg/m2	NVB 25 mg/m2 + DDP 80 mg/m2	(14/21)×(2)	①②③
Liang et al. (2005)	51//16	34/33	53.5/51.6′	≥60	Ⅲb, Ⅳ	Lac, LSCC, LASC	ADI 50 ml + NVB 25 mg/m2 + DDP 30mg/m2	NVB 25 mg/m2 + DDP 30 mg/m2	(21/28)×(3)	①③
Hu and Sun (2005)	65/22	45/42	38–76	≥60	Ⅲb, Ⅳ	Lac, LSCC, LASC	ADI 50 ml + NVB 25 mg/m2 + DDP 40 mg/m2	NVB 25 mg/m2 + DDP 40 mg/m2	56/(21 × 2)	①②
Zhang et al. (2006)	32/24	28/28	32–76	≥70	Ⅲb, Ⅳ	Lac, LSCC, LASC	ADI 50 ml + NVB 25 mg/m2 + DDP 30mg/m2	NVB 25 mg/m2 + DDP 30 mg/m2	14/(28×3–4)	①②③
Xing et al. (2014)	74/46	60/60	62–78/64–77	>60	Ⅲ, Ⅳ	NR	ADI 50 ml + NVB 25 mg/m2 + DDP 80 mg/m2	NVB 25 mg/m2 + DDP 80 mg/m2	14/(21 × 2)	①③④
Huang et al. (2008)	32/28	30/30	55–78	>60	Ⅲ, Ⅳ	NR	ADI 50 ml + NVB 25 mg/m2 + DDP 30 mg/m2	NVB 25mg/m2 + DDP 30 mg/m2	(14/28)×(2)	①②③④
Dai and Kong (2008)	32/28	30/30	50–70	>60	Ⅲ, Ⅳ	NR	ADI 80 ml + NVB 25 mg/m2 + DDP 30 mg/m2	NVB 25 mg/m2 + DDP 30 mg/m2	10/(28 × 2)	①②③
Zhang (2012)	124/180	168/136	34–72	>60	Ⅲ, Ⅳ	Lac, LSCC, LASC	ADI 60 ml + NVB 30 mg/m2 + DDP 120 mg/m2	NVB 30 mg/m2 + DDP 120 mg/m2	(14/28)×(2–3)	①③
Xu et al. (2007)	NR	36/36	35–72	≥60	Ⅲ, Ⅳ	NR	ADI 50 ml + NVB 25 mg/m2 + DVP 40 mg	NVB 25 mg/m2 + DDP 40 mg	(28/28)×(3)	①③
Chen (2008)	29/23	26/26	48–68/49–61	≥60	Ⅲ, Ⅳ	Lac, LSCC, LASC	ADI 50 ml + NVB 30 mg/m2 + DDP 25 mg/m2	NVB 30 mg/m2 + DDP 25 mg/m2	(10–15/21)	①②③
Yang et al. (2017)	69/47	58/58	41–77/41–77	>60	Ⅲ, Ⅳ	Lac, LSCC, LASC	ADI 60–80 ml + NVB 25 mg/m2 + DDP 30 mg/m2	NVB 25 mg/m2 + DDP 30 mg/m2	30/(21 × 2)	①③
Lin et al. (2007)	54/26	40/40	26–77	>60	Ⅲ, Ⅳ	Lac, LSCC	ADI 50 ml + NVB 25 mg/m2 + DDP 30 mg	NVB 25 mg/m2 + DDP 30 mg	8–10/(21 × 2)	①②③④
Shi ZC 2010	29/11	20/20	59/56′	50–80	Ⅲ, Ⅳ	Lac, LSCC	ADI 50 ml + NVB 25 mg/m2 + DDP 30 mg/m2	NVB 25 mg/m2 + DDP 30 mg/m2	(10/21)×(2)	①②④
Li et al. (2005a)	58/18	36/40	26–78/28–81	50–80	Ⅲ, Ⅳ	Lac, LSCC, LASC	ADI 40–80 ml + NVB 25 mg/m2 + DDP 20 mg/m2	NVB 25 mg/m2 + DDP 20 mg/m2	(22/21)×(2)	①②③
Su et al. (2005)	48/38	44/42	24–72	>50	Ⅲ, Ⅳ	Lac, LSCC, LASC	ADI 50 ml + NVB 25 mg/m2 + DDP 80 mg/m2	NVB 25 mg/m2 + DDP 80 mg/m2	(14/21–28)×(2)	①③
Wang et al. (2004)	67/31	49/49	35–72/50–80	50–80	Ⅲ, Ⅳ	Lac, LSCC, LASC	ADI 60–80 ml + NVB 25 mg/m2 + DDP 40 mg/m2	NVB 25mg/m2 + DDP 40mg/m2	(8–10/21–28)×(2)	①②③④
Wu et al. (2005)	48/12	32/28	59–76	>50	Ⅲ, Ⅳ	Lac, LSCC, LASC	ADI 50 ml + NVB 25 mg/m2 + DDP 100 mg/m2	NVB 25mg/m2 + DDP 100mg/m2	(30/21)×(2)	①②③④
Li and Cao (2007)	37/15	28/24	45–70/43–69	50–80	Ⅲ, Ⅳ	Lac, LSCC	AI 60 ml + NVB 40 mg + DDP20 mg	NVB 40 mg + DDP20 mg	20/(21–28*2)	①②③
Li (2010)	46/18	32/32	33–70/39–74	>60	Ⅲb, Ⅳ	Lac, LSCC, LCLC	CKSI 20 ml + NVB25 mg/m2 + DDP 30 mg/m2	NVB25 mg/m2 + DDP 30 mg/m2	8/(21 × 2)	①②
Song (2010)	23/24	27/20	36–68/35–69	≥70	Ⅲb, Ⅳ	Lac, LSCC	CKSI 20 ml + NVB 25 mg/m2 + DDP 80 mg/m2	NVB 25 mg/m2 + DDP 80 mg/m2	21 × 2	①②③
Sang (2012)	65/39	54/50	32–73/31–72	≥70	Ⅲb, Ⅳ	Lac, LSCC	CKSI 20 ml + NVB 25 mg/m2 + DDP 25 mg/m2	NVB 25 mg/m2 + DDP 25 mg/m2	14 × 2	①②③
Wu et al. (2006)	50/37	43/44	33–76	>60	Ⅲ, Ⅳ	Lac, LSCC, LASC	CKSI 20 ml + NVB 30 mg/m2 + DDP30 mg/m2	NVB 30 mg/m2 + DDP30 mg/m2	(10×(3–6))/(21×(3–6))	①②③
Su and Li (2007)	46/34	50/30	35–70/37–72	>60	Ⅲ, Ⅳ	Lac, LSCC	CKSI 20 ml + NVB 25mg/m2 + DDP 75mg/m2	NVB 25 mg/m2 + DDP 75 mg/m2	(14/(21–28))×2	①②③
Wang and Feng (2010)	65/15	40/40	45–72/43-7L	≥60	Ⅲa, Ⅲb, Ⅳ	Lac, LSCC, O	CKSI 30 ml + NVB 25 mg/m2 + DDP 30 mg	NVB 25 mg/m2 + DDP 30 mg	21 × 2	①②③
Guo et al. (2007)	38/25	32/31	43–71	>50	Ⅲ, Ⅳ	Lac, LSCC, LCLC, O	CKSI 20 ml + NVB 25 mg/m2 + DDP 20 mg/m2	NVB 25 mg/m2 + DDP 20 mg/m2	21 × 2	①②③
Wu and Chen (2014)	22/24	23/23	27–72/32–70	≥60	Ⅲa, Ⅲb, Ⅳ	Lac, LSCC, LASC	CKSI 20 ml + NVB 25 mg/m2 + DDP 80 mg/m2	NVB 25 mg/m2 + DDP 80 mg/m2	((10–15)/21)×2	①②③
Zhao et al. (2006)	NR	47/46	56′	≥60	Ⅲb, Ⅳ	Lac, LSCC, LASC	CSI 20 ml + NVB 25mg/m2 + DDP 40–50 mg	NVB 25mg/m2 + DDP 40–50 mg	(8–10/28)*2	①②④
Ma et al. (2005)	44/18	32/30	32–70/34–70	≥70	Ⅲ, Ⅳ	Lac, LSCC	DLSI 40–60 ml + NVB 25mg/m2 + DDP 80–100mg/m2	NVB 25 mg/m2 + DDP 80–100 mg/m2	45/(21 × 2)	①②③
Huang et al. (2011)	28/22	25/25	35～72	≥60	Ⅲa, Ⅲb, Ⅳ	Lac, LSCC	DLSI 40 ml + NVB 25mg/m2 + DDP 50mg/m2	NVB 25 mg/m2 + DDP 50 mg/m2	(21/28)×2	①②③
Cao (2009)	28/22	25/25	40–75	≥60	Ⅲb, Ⅳ	Lac, LSCC	HCSI 20 ml + NVB 40 mg + DDP 40 mg	NVB 40 mg + DDP 40 mg	21*3	①
Yang and Xi (2006)	38/22	30/30	35–69	≥70	Ⅲ, Ⅳ	Lac, LSCC, LASC	HCSI 15 ml + NVB 25 mg·kg-1·(m2)-1 + DDP 40 mg·kg-1·(m2)-1	NVB 25 mg·kg-1·(m2)-1 + DDP 40 mg·kg-1·(m2)-1	21*2	①②③
Miao et al. (2007)	50/37	43/44	34–74/34–72	＞60	Ⅲ, Ⅳ	Lac, LSCC, LASC	HCSI 20 ml + NVB 30 mg/m2 + DDP 30 mg/m2	NVB 30 mg/m2 + DDP 30 mg/m2	5*(3–6)	①②③
Dong et al. (2009)	42/26	34/34	60–79/62–78	72/74	Ⅲa, Ⅲb, Ⅳ	Lac, LSCC	JOEI 30 ml + NVB 30 mg/m2 + DDP 50 mg/m2	NVB 30 mg/m2 + DDP 50 mg/m2	14/(21 × 4)	①②③④
Chen and Cai (2007)	41/19	30/30	35–72/37–73	50–90	Ⅲ, Ⅳ	Lac, LSCC, LASC, O	10%JOEI 20 ml + NVB 25 mg + DDP 25–30 mg	NVB 25 mg + DDP 25–30 mg	8–10/((21–28)×4)	①②③④
Liao (2016)	62/18	40/40	33–74	NR	Ⅲa, Ⅲb, Ⅳ	Lac, LSCC	JOEI 40 ml + NVB 25 mg/m2 + DDP 80 mg/m2	NVB 25mg/m2 + DDP 80mg/m2	21 × 2	①②③
Li and Wu (2010)	27/9	20/16	32–72	NR	Ⅲa, Ⅲb, Ⅳ	Lac, LSCC, LCLC	JOEI 100 ml + NVB 25 mg/m2 + DDP 25 mg/d	NVB 25 mg/m2 + DDP 25 mg/d	21 × 2	①②③
Ren et al. (2012)	109/45	78/76	51.86 + 3.26/52.21 + 2.72*	>60	Ⅲ, Ⅳ	NR	JOEI 40 ml + NVB 25 mg/m3 + DDP 70 mg/m3	NVB 25 mg/m3 + DDP 70 mg/m3	30 × 2/21 × 3	①③
Wu (2008)	48/12	32/28	18–78	≥60	Ⅲb, Ⅳ	NR	JOEI 30 ml + NVB 25 mg/m2 + DDP 30 mg/m2	NVB 25 mg/m2 + DDP 30 mg/m2	15/(21 × 2)	①②③
Du and Shi (2006)	76/37	56/57	27–72	≥70	Ⅲb, Ⅳ	Lac, LSCC, LASC, LCLC	JOEI 30 ml + NVB 30 mg/m2 + DDP 50 mg/NVB 30 mg/m2 + DDP50 mg × 2/3	NVB 30 mg/m2 + DDP 50 mg/NVB 30 mg/m2+DDP50 mg × 2/3	(14/21)×4	①③④
Wang et al. (2011)	42/30	36/36	32–75	>60	Ⅲb, Ⅳ	Lac, LSCC	JOEI 50–100 ml + NVB 25 mg/m2 + DDP 30 mg/m2	NVB 25 mg/m2 + DDP 30 mg/m2	30/(21 × 52)	①②③④
Deng (2014)	26/26	26/26	43–70/45–70	≥60	Ⅲ, Ⅳ	Lac, LSCC, LASC	KAI 60 ml + NVB 25 mg/m2 + DDP 20 mg/m2	NVB 25 mg/m2 + DDP 20 mg/m2	(14 × 2)/((21 × 8)×2)	①②③
Huang et al. (2006)	34/16	25/25	35–75/36–78	≥60	Ⅲb, Ⅳ	Lac, LSCC	KAI 20–30 ml + NVB 25 mg/m2 + DDP 25mg/m2	NVB 25 mg/m2 + DDP 25 mg/m2	21 × 2	①②③
Huang (2011)	194/92	144/142	43–70/42–71	≥60	Ⅲ, Ⅳ	Lac, LSCC, LASC, LCLC	KAI 60 ml + NVB 25 mg/m2 + DDP 30 mg/m2	NVB 25 mg/m2 + DDP 30 mg/m2	(14/28)×4	①②③
Zou (2013)	52/24	38/38	53–71/52–74	≥60	Ⅲ, Ⅳ	Lac, LSCC	KAI 40 ml + NVB 25 mg/m2 + DDP 30 mg/m2	NVB 25 mg/m2 + DDP 30 mg/m2	21 × 2	①②③
Zhang and Luan (2005)	43/19	30/32	35–74/37–76	>60	Ⅲ, Ⅳ	Lac, LSCC	KAI 40–60 ml + NVB 25 mg/m2 + DDP 40 mg/m2	NVB 25 mg/m2 + DDP 40 mg/m2	((8–10)/(21–28))×2	①②③④
Yin (2007)	41/30	31/40	35–80/40–78	>70	Ⅲb, Ⅳ	Lac, LSCC, LASC	KAI 40 ml + NVB 25 mg/m2 + DDP 100 mg/m2	NVB 25 mg/m2 + DDP 100 mg/m2	7 × 2	①③
Ran et al. (2009)	86/42	22/62	54/55′	NR	Ⅲ, Ⅳ	NR	KAI 30 ml + NVB 25 mg/m2 + DDP 100 mg/m2	NVB 25 mg/m2 + DDP 100 mg/m2	28	①③
Huang et al. (2013)	52/44	48/48	42–71	NR	Ⅲb, Ⅳ	Lac, LSCC, LASC	KLTI 100–200 ml + NVB 25 mg/m2 + DDP 80 mg/m2	NVB 25 mg/m2 + DDP 80 mg/m2	14/(21*2)	①②③
Zhang and Qi (2010)	48/14	30/32	38–71/40–70	60–85	Ⅲb, Ⅳ	Lac, LSCC, LASC	KLTI 200 ml/d + NVB 25 mg/m2 + DDP 40 mg	NVB 25 mg/m2 + DDP 40 mg	21*2	①②③
Wang and Zhang (2007)	62/18	39/41	38–71/40–70	60–85	Ⅲb, Ⅳ	Lac, LSCC, LASC	KLTI 200 ml/d + NVB 25 mg/m3 + DDP 40 mg	NVB 25 mg/m3 + DDP 40 mg	21*2	①②③
Hou (2008)	41/27	34/34	37–71/35–69	≥70	Ⅲb, Ⅳ	Lac, LSCC	KLTI 100 ml + NVB 25 mg/m2 + DDP 30 mg/m2	NVB 25mg/m2 + DDP 30 mg/m2	21*2	①②③
Li and Dong (2016)	43/35	39/39	35–72/36–71	NR	Ⅲ, Ⅳ	Lac, LSCC, LASC	KLTI 200 ml + NVB 25 mg/m2 + DDP 30 mg/m2	NVB 25 mg/m2 + DDP 30 mg/m2	20	①②③
Xie et al. (2003)	49/38	43/44	38–76	NR	Ⅲ, Ⅳ	Lac, LSCC, LASC	KLTI 100 ml + NVB 25 mg/m2 + DDP 75 mg/m2	NVB 25 mg/m2 + DDP 75 mg/m2	30*1	①②③
Lv et al. (2004)	41/19	30/30	29–67	≥70	Ⅲ, Ⅳ	Lac, LSCC, LASC	KLTI 100 ml + NVB 25 mg/kg/m2 + DDP 40 mg/kg/m2	NVB 25 mg/kg/m2 + DDP 40 mg/kg/m2	21	①②
Xu and Li (2010)	28/17	23/22	70–79	≥60	Ⅲ, Ⅳ	Lac, LSCC	KLTI 100 ml + NVB 25 mg/m2 + DDP 30 mg/m2	NVB 25 mg/m2 + DDP 30 mg/m2	21	①②③
Li et al. (2018)	44/36	40/40	54.69 ± 4.94/55.37 ± 5.18*	NR	Ⅲ, Ⅳ	Lac, LSCC, LASC	KLTI 10 g·次−1 + NVB 25 mg·m−2 + DDP 80 mg·m−2	NVB 25 mg·m−2 + DDP 80 mg·m−2	21*2	①③
Liao et al., (2009)	176/112	144/144	62/58′	NR	Ⅲ, Ⅳ	Lac, LSCC, LCLC	KLTI 200 ml + NVB 25 mg/m2 + DDP 30 mg/m2	NVB 25 mg/m2 + DDP 30 mg/m2	20*2	①②③④
Wang and Han (2009)	35/25	30/30	35–75/35–74	>60	Ⅲ, Ⅳ	Lac, LSCC, LCLC	KLTI 200 ml + NVB 25 mg/m2 + DDP 25 mg/m2	NVB 25 mg/m2 + DDP 25 mg/m2	21*2	①②③④
Xie et al. (2003)	49/38	41/40	38–76	NR	Ⅲb, Ⅳ	Lac, LSCC, LASC, LCLC	KLTI 100 ml + NVB 25 mg/m2 + DDP 75 mg/m2/d	NVB 25 mg/m2 + DDP 75 mg/m2/d	21/30*2	②
Liu and Huang (2011)	46/14	30/30	50–65/48–63	≥70	Ⅲ, Ⅳ	Lac, LSCC	SFI 60 ml + NVB 25 mg/m2 + DDP 30 mg/m2	NVB 25 mg/m2 + DDP 30 mg/m2	14*2	②
Zhao et al. (2017)	61/39	50/50	52–69/51–67	≥60	Ⅲ, Ⅳ	Lac, LSCC, LCLC	SFI 60 ml + NVB 25 mg/m2 + DDP 30 mg/m2	NVB 25 mg/m2 + DDP 30 mg/m2	42/(21*3)	①②
Chen (2010)	31/16	23/24	48–76/50–75	≥60	Ⅲ, Ⅳ	Lac, LSCC, LCLC	SFI 50 ml + NVB 25 mg/m2 + DDP 30 mg/m2	NVB 25 mg/m2 + DDP 30 mg/m2	42/(21*3)	①③
Li (2014)	37/23	30/30	53–65/55–67	≥60	Ⅲ, Ⅳ	Lac, LSCC, LCLC	SFI 50 ml + NVB 25 mg/m2 + DDP 30 mg/m2	NVB 25 mg/m2 + DDP 30 mg/m2	42/(21*3)	①②
Lu et al. (2010)	43/17	30/30	38–70/40–69	＞60	Ⅲ, Ⅳ	Lac, LSCC, LCLC	SFI 60 ml + NVB 25 mg/m2 + DDP 30 mg/m2	NVB 25 mg/m2 + DDP 30 mg/m2	10*2	①③④
Wu et al. (2007)	24/18	42/42	NR	NR	Ⅲ, Ⅳ	Lac, LSCC, LCLC	SFI 60 ml + NVB 30 mg/m2 + DDP 80 mg/m2 NVB 25 mg/m2 + DDP 30 mg/m2	NVB 25 mg/m2 + DDP 30 mg/m2 SFI 60 ml + NVB 30 mg/m2 + DDP 80 mg/m2	10/(21*2)	④
Wu et al. (2006)	24/18	42/42	54.0 ± 9.99/54.63 ± 10.74*	NR	Ⅲ, Ⅳ	Lac, LSCC, LCLC	SFI 60 ml + NVB 30 mg/m2 + DDP 80 mg/m2 NVB 25 mg/m2 + DDP 30 mg/m2	NVB 25 mg/m2 + DDP 30 mg/m2 SFI 60 ml + NVB 30 mg/m2 + DDP 80 mg/m2	10/(21*2)	③
Gong and Luo (2008)	41/19	30/30	65–79/66–80	≥50	Ⅲb, Ⅳ	Lac, LSCC	SFI 50 ml + NVB 25 mg/m2 + DDP 25 mg/m2	NVB 25mg/m2 + DDP 25mg/m2	21*2	①③
Zhang (2008b)	65/19	42/42	56 ± 8.2/57 ± 7.9*	≥60	Ⅲb, Ⅳ	Lac, LSCC, LCLC	SFI 60 ml + NVB 25 mg/m2 + DDP 80 mg/m2	NVB 25mg/m2 + DDP 80mg/m2	21*3	①②③
Chen et al., (2004)	53/29	42/40	39–74/29–75	NR	Ⅲb, Ⅳ	Lac, LSCC, LASC, O	SGMI 60–100 ml + NVB 25 mg/m2 + DDP 60–80 mg/m2	NVB 25mg/m2 + DDP 60–80 mg/m2	21*2	①③
Zhang (2008a)	45/21	32/34	49–78/50–77	≥50	Ⅲb, Ⅳ	Lac, LSCC, LASC	SGMI 60 ml + NVB 25 mg/m2 + DDP 40 mg/m2	NVB 25 mg/m2 + DDP 40 mg/m2	(21–28)*1	①②③
Chen et al., (2011)	42/18	30/30	46–69/47–69	≥60	Ⅲb, Ⅳ	Lac, LSCC	SGMI 60 ml + NVB 25 mg/m2 + DDP 80 mg/m2	NVB 25 mg/m2 + DDP 80 mg/m2	15*2	①②③
Zhou and Li (2003)	39/21	30/30	28–69/35–62	≥60	Ⅲ, Ⅳ	NR	SGMI 50 ml + NVB 35 mg/m2 + DDP 25 mg/m2	NVB 35 mg/m2 + DDP 25 mg/m2	(10/21)*2	①②③
Yang et al. (2012)	50/10	30/30	35–67/32–66	≥60	Ⅲ, Ⅳ	Lac, LSCC, LCLC	SGMI 40 ml + NVB 25 mg/m3 + DDP 30–40 mg/m2	NVB 25 mg/m3 + DDP 30–40 mg/m2	14*2	①②③
Li et al. (2005)	40/10	30/30	38–76/38–75	≥50	Ⅲb, Ⅳ	Lac, LSCC, LASC	SMI 40–60 ml + NVB 40 mg + DDP 30 mg/m2	NVB 40 mg + DDP 30 mg/m2	21×(2–3)	①②③
Hu et al. (2008)	48/32	40/40	61.2 ± 5.3/60.5 ± 4.3*	<70	Ⅲ, Ⅳ	Lac, LSCC, LASC	SMI 40 ml + NVB 25 mg/m2 + DDP 20 mg	NVB 25 mg/m2 + DDP 20 mg	28 × 2	①④
Bian et al., (2005)	45/15	30/30	39–71/37–70	50–80	Ⅲ, Ⅳ	NR	SMI 40–60 ml + NVB25 mg/m2 + DDP 80 mg/m2	NVB25 mg/m2 + DDP 80 mg/m2	(14/21)×2	①②③
Shen et al., (2012)	50/10	30/30	45–72/43–70	≥60	Ⅲa, Ⅲb, Ⅳ	Lac, LSCC	SMI 60 ml + NVB 25 mg/m2 + DDP 4 mg/m2	NVB 25 mg/m2 + DDP 4 mg/m2	(14 × 2)/(21 × 2)	①②③
Zhang et al., (2013)	36/18	27/27	45–71/47–69	≥60	Ⅲ, Ⅳ	Lac, LSCC, LASC	SMI 60 ml + NVB 25 mg/m2 + DDP 25 mg/m2	NVB 25 mg/m2 + DDP 25 mg/m2	14 × 2	③
Xue (2007)	45/15	30/30	26–75/34–78	≥60	Ⅲ, Ⅳ	NR	SMI 60 ml + NVB 35 mg/m2 + DDP 25 mg/m2	NVB 35 mg/m2 + DDP 25 mg/m2	(10/21)×2	①②③
Chen et al. (2010)	51/9	30/30	29–65/26–64	>60	Ⅲa, Ⅲb, Ⅳ	Lac, LSCC	SMI 60 ml + NVB 25 mg/m2 + DDP 30 mg/m2	NVB 25 mg/m2 + DDP 30 mg/m2	28 × 2	①②③
Hu et al. (2010)	38/22	30/30	22–74	NR	Ⅲ, Ⅳ	Lac, LSCC	SMI 60 ml + NVB 25 mg/m2 + DDP 80 mg/m2	NVB 25 mg/m2 + DDP 80 mg/m2	21 × 2	①②③
Lu et al. (2011)	49/11	30/30	46–64/40–62	>60	Ⅲa, Ⅲb, Ⅳ	Lac, LSCC	SMI 50 ml + NVB 25 mg/m2 + DDP 30 mg/m2	NVB 25 mg/m2 + DDP 30 mg/m2	(14 × 2)/(28 × 2)	②③
Geng (2004)	25/15	25/15	25–64/25–68	≥70	Ⅲ, Ⅳ	NR	SQFZI 250 ml + NVB 25 mg/m2 + DDP 80 mg/m2	NVB 25 mg/m2 + DDP 80 mg/m2	21	①②③④
Li et al. (2007)	65/22	44/43	42–81	≥60	Ⅲ, Ⅳ	Lac, LSCC, LCLC, O	SQFZI 250 ml + NVB 0.5 mg/m2 + DDP 40 mg/m2	NVB 0.5 mg/m2 + DDP 40 mg/m2	28*4	①③
Shi et al. (2007)	40/19	32/27	37–69	≥60	Ⅲb, Ⅳ	Lac, LSCC	SQFZI 250 ml + NVB 30 mg/m2 + DDP 80 mg/m2	NVB 30 mg/m2 + DDP 80 mg/m2	15/(21*2)	①②③
Wang et al. (2007)	26/10	18/18	38–75/34–73	≥50	Ⅲb, Ⅳ	Lac, O	SQFZI 250 ml + NVB 25 mg/m2 + DDP 60 mg/m2	NVB 25 mg/m2 + DDP 60 mg/m2	8*3	①②③
Yu (2007)	43/19	30/32	35–74/37–76	≥60	Ⅲ, Ⅳ	Lac, LSCC	SQFZI 250 ml + NVB 25 mg/m2 + DDP 40 mg/m2	NVB 25 mg/m2 + DDP 40 mg/m2	(8–10)*4	①②③④
Zhao et al. (2007)	51/18	35/34	61–82	>60	Ⅲa, Ⅲb, Ⅳ	Lac, LSCC, LCLC, O	SQFZI 250 ml + NVB 0.5 mg/m2 + DDP 30 mg/m2	NVB 0.5 mg/m2 + DDP 30 mg/m2	10/[(21–28)*(2–3)]	①③
Jiang and Zhang (2008)	29/11	20/20	32–71/35–69	NR	Ⅲb, Ⅳ	Lac, LSCC	SQFZI 250 ml + NVB 25 mg/m2 + DDP 25 mg/m2	NVB 25 mg/m2 + DDP 25 mg/m2	21*2	③
Lv (2008)	65/15	40/40	52–78/51–78	≥50	Ⅲb, Ⅳ	Lac, LSCC, LASC	SQFZI 250 ml + NVB 25 mg/m2 + DDP 30 mg/m2	NVB 25 mg/m2 + DDP 30 mg/m2	21*2	①②③
Li et al. (2010)	56/29	43/42	47–72/45–75	≥60	Ⅲb, Ⅳ	Lac, LSCC, LASC	SQFZI 250 ml + NVB 35 mg/m2 + DDP 80 mg/m2/DDP 35 mg/m2	NVB 35 mg/m2 + DDP 80 mg/m2/DDP 35 mg/m2	14/(21*2)	②③④
Lu (2010)	44/16	30/30	(65.2 ± 1.5)/(65.3 ± 1.4)*	≥50	Ⅲb, Ⅳ	Lac, LSCC	SQFZI 250 ml + NVB 25 mg/m2 + DDP 40 mg/m2	NVB 25 mg/m2 + DDP 40 mg/m2	10*2	②③
Miao et al. (2010)	60/19	38/41	38–71/40–70	≥60	Ⅲb, Ⅳ	Lac, LSCC, LASC	SQFZI 250 ml + NVB 25 mg/m2 + DDP 30 mg/m2	NVB 25mg/m2 + DDP 30mg/m2	21*2	①②③
Wang and Guo (2009)	35/21	28/28	32–75	>50	Ⅲb, Ⅳ	Lac, LSCC	XAPI 40–60 ml + NVB 25 mg/m2 + DDP 30 mg/m2	NVB 25mg/m2 + DDP 30mg/m2	15/(21 × 2)	①②③④

Note: M, Male; F, Female; T, Treatment group; C, Control group; ADI, Aidi injection; AI, *Astragalus* injection; CSI, Chansu injection; CKSI, Compound kushen injection; DLSI, Delisheng injection; HCSI, Huachansu injection; JOEI, Javanica oil emulsion injection; KAI, Kangai injection; KLTI, Kanglaite injection; NP, vinorelbine plus cisplatin; SFI, Shenfu injection; SMI, Shenmai injection; SQFZI, Shenqifuzheng injection; SGMI, Shengmai injection; XAPI, Xiaoaiping injection; ① Clinical effective rate; ② Performance status; ③ Leukopenia; ④ hemoglobin reduction; ⑤ thrombocytopenia; ⑥ gastrointestinal reactions.

### Methodological Quality

**FIGURE 4:**
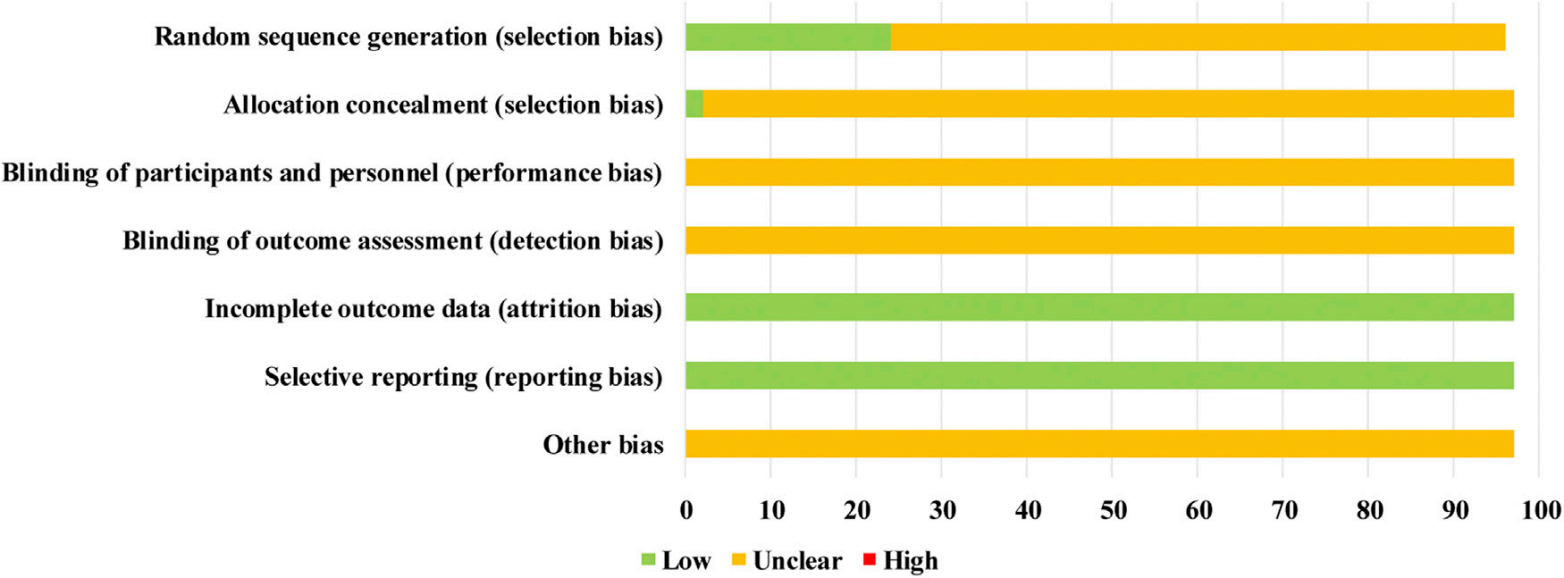
Risk of bias graph.

The quality of the RCTs was evaluated by the Cochrane risk of bias tool. Regarding selection bias, 24 of 97 RCTs were rated as “low risk” because 21 RCTs adopted random number tables and three RCTs used lot-drawing for randomization. Three of the included RCTs described allocation concealment by the envelope method. The risk of the remaining RCTs was deemed “unclear”. Not all provided information on the blinding methods, so the performance bias and detection bias were assessed as “unclear”. In addition, since all RCTs did not have incomplete data and selective reporting, their attrition bias and reporting bias were evaluated as “low risk”. For the other bias assessment of the quality of the RCTs, the original studies did not mention the details about other problems that were relative to a high risk of bias; hence, the other bias was remarked as “unclear”. Overall, the quality of the enrolled RCTs was not high, and specific information about the risk of bias is shown in Figure 4.

### Network Meta-analysis

This study conducted a direct comparison and subgroup analysis on the six outcomes involved. The three outcomes (performance status, leukopenia, and gastrointestinal reaction) with significant heterogeneity in the subgroup analysis were further analyzed by meta regression. Then three factors of age, DDP dose and cancer grade were explored relationship with heterogeneity. The tumor stages included in this study were all stage Ⅲ-Ⅳ, which were consistent and had no obvious clinical heterogeneity. Therefore, this study only performed regression analysis on age and DDP dose. The regression results showed that age was not a source of heterogeneity. The dose of the chemotherapy drug DDP was one of the sources of heterogeneity, which could explain part of the inter-study variation (Adj R-squared = 8.55%) (The details were shown in the Supplementary material Presentation File 1.

#### Clinical Effectiveness Rate

A total of 85 RCTs with 14 types of CHIs reported information on the clinical effectiveness rate. The results suggested that six types of CHIs: Aidi injection, Compound kushen injection, Huachansu injection, Kangai injection, Kanglaite injection, and Shenmai injection, in combination with NP presented higher clinical effectiveness rates than NP alone. In addition, significant differences were detected across these five types of CHIs compared with NP (Table 2).

**TABLE 2:** Results of the network meta-analysis of the clinical effectiveness rate (upper right quarter) and the performance status (lower left quarter).

ADI + NP	0.72 (0.57.0.89)	0.72 (0.22.2.15)	1.41 (0.94.2.12)	1.057 (0.41.2.66)	1.39 (0.66.3.089)	1.46 (0.77.2.72)	0.91 (0.62.1.33)	1.075 (0.74.1.56)	1.41 (1.00.1.99)	0.87 (0.55.1.39)	0.90 (0.51.1.58)	1.33 (0.83.2.13)	1.00 (0.65.1.55)	1.13 (0.31.3.98)
**0.27 (0.19,0.38)**	**NP**	1.005 (0.31.2.94)	**1.95 (1.40,2.78)**	1.48 (0.58.3.59)	1.93 (0.94.4.17)	**2.025 (1.11,3.68)**	1.26 (0.93.1.75)	**1.50 (1.091,2.07)**	**1.95 (1.49,2.58)**	1.22 (0.82.1.80)	1.25 (0.74.2.10)	**1.84 (1.23,2.76)**	1.39 (0.95.2.021)	1.57 (0.44.5.48)
0.51 (0.12.2.41)	1.92 (0.48.8.59)	**AI + NP**	1.93 (0.62.6.45)	1.44 (0.36.6.22)	1.93 (0.50.7.99)	1.99 (0.58.7.099)	1.24 (0.42.4.14)	1.49 (0.48.5.045)	1.94 (0.64.6.44)	1.19 (0.39.4.20)	1.25 (0.38.4.29)	1.84 (0.57.6.13)	1.39 (0.44.4.79)	1.56 (0.31.8.99)
0.79 (0.44.1.44)	**2.99 (1.87,4.82)**	1.57 (0.32.6.74)	**CKSI + NP**	0.74 (0.29.1.98)	0.99 (0.45.2.35)	1.026 (0.52.2.076)	0.65 (0.41.1.036)	0.77 (0.47.1.23)	1.00 (0.64.1.56)	0.62 (0.37.1.052)	0.64 (0.35.1.19)	0.95 (0.55.1.58)	0.71 (0.42.1.17)	0.79 (0.21.3.20)
0.84 (0.25.3.012)	3.15 (0.9810.84)	1.66 (0.25.10.1)	1.056 (0.30.3.95)	**CSI + NP**	1.31 (0.43.4.15)	1.37 (0.47.4.28)	0.86 (0.33.2.34)	1.013 (0.39.2.75)	1.32 (0.52.3.45)	0.82 (0.31.2.33)	0.85 (0.31.2.47)	1.24 (0.48.3.46)	0.94 (0.37.2.55)	1.087 (0.21.4.83)
0.69 (0.23.1.94)	2.57 (0.97.6.93)	1.35 (0.22.7.53)	0.85 (0.29.2.63)	0.81 (0.17.3.86)	**DLSI + NP**	1.047 (0.37.2.71)	0.66 (0.28.1.42)	0.77 (0.34.1.66)	1.015 (0.45.2.18)	0.63 (0.27.1.44)	0.65 (0.25.1.56)	0.96 (0.39.2.18)	0.72 (0.31.1.606)	0.80 (0.19.3.60)
0.72 (0.30.1.78)	2.71 (1.21,6.28)	1.41 (0.27.7.14)	0.91 (0.35.2.40)	0.86 (0.20.3.66)	1.054 (0.30.3.75)	**HCSI + NP**	0.63 (0.32.1.23)	0.74 (0.38.1.45)	0.96 (0.50.1.88)	0.60 (0.30.1.22)	0.62 (0.28.1.37)	0.93 (0.45.1.86)	0.70 (0.33.1.37)	0.77 (0.20.3.24)
**2.026 (1.016,4.052)**	7.57 (4.25,13.79)	3.97 (0.85.17.81)	2.53 (1.17,5.55)	2.40 (0.601.8.93)	2.94 (0.94.8.94)	**2.80 (1.011,7.55)**	**JOEI + NP**	1.18 (0.75.1.89)	**1.54 (1.01,2.35)**	0.96 (0.57.1.59)	0.99 (0.54.1.82)	1.46 (0.88.2.40)	1.098 (0.67.1.77)	1.23 (0.34.4.63)
0.75 (0.381.49)	**2.80 (1.59,5.084)**	1.46 (0.30.6.62)	0.93 (0.44.2.01)	0.89 (0.22.3.30)	1.082 (0.35.3.31)	1.034 (0.37.2.84)	**0.37 (0.16,0.84)**	**KAI + NP**	1.30 (0.87.1.98)	0.82 (0.49.1.35)	0.84 (0.45.1.52)	1.23 (0.72.2.071)	0.93 (0.57.1.51)	1.048 (0.28.3.82)
0.67 (0.40.1.14)	**2.54 (1.74,3.70)**	1.32 (0.28.5.44)	0.85 (0.47.1.54)	0.80 (0.22.2.72)	0.98 (0.34.2.81)	0.94 (0.37.2.26)	**0.34 (0.16,0.67)**	0.91 (0.44.1.79)	**KLTI + NP**	0.62 (0.39.1.004)	0.64 (0.35.1.16)	0.94 (0.58.1.56)	0.71 (0.44.1.14)	0.81 (0.22.2.86)
0.47 (0.21.1.048)	1.75 (0.85.3.68)	0.91 (0.18.4.45)	0.59 (0.25.1.42)	0.55 (0.13.2.22)	0.69 (0.20.2.25)	0.65 (0.21.1.94)	**0.23 (0.091,0.59)**	0.63 (0.25.1.58)	0.69 (0.31.1.57)	**SFI + NP**	1.036 (0.54.1.98)	1.52 (0.86.2.68)	1.14 (0.65.2.00)	1.30 (0.34.5.45)
0.64 (0.31.1.33)	**2.40 (1.27,4.61)**	1.25 (0.26.5.75)	0.80 (0.37.1.79)	0.76 (0.19.2.89)	0.93 (0.28.3.03)	0.88 (0.31.2.54)	**0.32 (0.13,0.76)**	0.86 (0.36.2.045)	0.95 (0.45.2.01)	1.37 (0.51.3.61)	**SGMI + NP**	1.48 (0.75.2.80)	1.11 (0.58.2.072)	1.25 (0.31.4.85)
0.88 (0.45.1.82)	**3.33 (1.84,6.10)**	1.74 (0.356.7.77)	1.11 (0.52.2.43)	1.063 (0.27.3.95)	1.29 (0.41.4.13)	1.23 (0.44.3.36)	0.44 (0.19.1.013)	1.19 (0.51.2.74)	1.32 (0.65.2.68)	1.91 (0.73.4.84)	1.38 (0.58.3.36)	SMI + NP	0.75 (0.43.1.31)	0.6 (0.22.3.15)
0.91 (0.51.1.59)	**3.38 (2.20,5.28)**	1.77 (0.37.7.45)	1.14 (0.60.2.17)	1.075 (0.29.3.77)	1.32 (0.45.3.83)	1.26 (0.49.3.14)	0.45 (0.21.0.92)	1.22 (0.58.2.48)	1.34 (0.75.2.39)	1.94 (0.82.4.61)	1.41 (0.65.3.086)	1.019 (0.48.2.14)	SQFZI + NP	1.13 (0.29.4.29)
1.53 (0.418.6.43)	**5.73 (1.63,23.26)**	3.023 (0.45.20.71)	1.92 (0.50.8.42)	1.83 (0.31.11)	2.23 (0.44.12.45)	2.10 (0.47.10.54)	0.76 (0.19.3.34)	2.064 (0.50.9.33)	2.26 (0.60.9.56)	3.26 (0.78.16.42)	2.39 (0.60.11.06)	1.74 (0.42.7.98)	1.69 (0.44.7.34)	XAPI + NP

Note: The differences between the compared groups were deemed as significant when the 95% CI of the OR did not contain 1.00, which is marked as bold font. ADI, Aidi injection; AI, *Astragalus* injection; CSI, Chansu injection; CKSI, Compound kushen injection; DLSI, Delisheng injection; HCSI, Huachansu injection; JOEI, Javanica oil emulsion injection; KAI, Kangai injection; KLTI, Kanglaite injection; NP, vinorelbine plus cisplatin; SFI, Shenfu injection; SMI, Shenma injection; SQFZI, Shenqifuzheng injection; SGMI, Shengmai injection; XAPI, Xiaoaiping injection.

**FIGURE 5:**
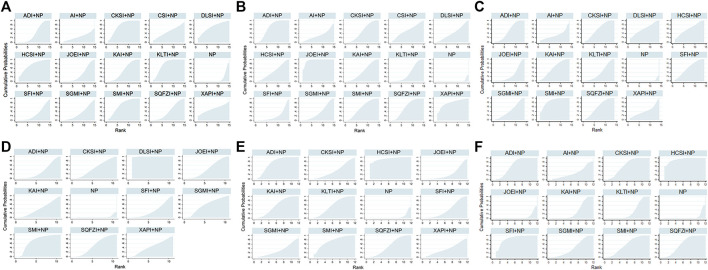
Plot of the surface under the cumulative ranking curves for outcomes. **(A)** Clinical effectiveness rate; **(B)** Performance status; **(C)** leukopenia; **(D)** hemoglobin reduction; **(E)** thrombocytopenia; **(F)** gastrointestinal reactions. Note: The surface under the cumulative ranking curve (SUCRA) was used to estimate the ranking probabilities for different CHIs, which ranged from 0% to 100%. A better treatment is indicated by a higher SUCRA value. NP, vinorelbine and cisplatin; ADI, Aidi injection; AI, *Astragalus* injection; CSI, Chansu injection; CKSI, Compound kushen injection; DLSI, Delisheng injection; HCSI, Huachansu injection; JOEI, Javanica oil emulsion injection; KAI, Kangai injection; KLTI, Kanglaite injection; SFI, Shenfu injection; SMI, Shenmai injection; SQFZI, Shenqifuzheng injection; SGMI, Shengmai injection; XAPI, Xiaoaiping injection.

Compared with Javanica oil emulsion injection plus NP, Kanglaite injection plus NP might hold greater potential for increasing the clinical effectiveness rate (Table 2). Figure 5A presents the cumulative probabilities (SUCRA values) that 14 types of CHIs combined with NP improve the clinical effectiveness rate. The combination of Kanglaite injection and NP was associated with the highest probability of being the best option for improving the clinical effectiveness rate (78.34%), followed by Compound kushen injection plus NP (77.55%) and Huachansu injection plus NP (76.08%). The SUCRA values of each intervention in terms of different outcomes are presented in Table 3.

**TABLE 3:** Ranking probability of various interventions.

Intervention	Clinical effectiveness rates		Performance status		Leukopenia		Hemoglobin		Thrombocytopenia		Gastrointestinal	
	SUCRA (%)	Rank	SUCRA (%)	Rank	SUCRA (%)	Rank	SUCRA (%)	Rank	SUCRA (%)	Rank	SUCRA (%)	Rank
CSI + NP	49.37	8	55.10	6	−	−	−	−	−	−	−	−
HCSI + NP	76.08	3	46.43	9	61.72	3	−	−	92.67	1	92.52	1
SMI + NP	71.74	4	60.48	5	86.87	1	76.26	2	74.17	3	62.15	6
DLSI + NP	70.65	5	44.09	10	61.30	5	99.54	1	−	−	−	−
CKSI + NP	77.55	2	52.87	7	61.71	4	50.60	5	40.09	8	68.01	3
SQFZI + NP	43.40	9	62.56	4	61.74	2	55.06	4	54.64	6	64.56	5
XAPI + NP	53.03	6	81.01	2	40.54	11	47.26	6	39.94	9	−	−
KLTI + NP	78.34	1	39.76	11	40.77	10	−	−	59.89	4	32.75	9
ADI + NP	43.13	10	70.37	3	54.33	8	35.04	9	76.20	2	67.97	4
KAI + NP	50.90	7	47.84	8	61.06	6	42.40	8	59.36	5	33.28	8
AI + NP	26.97	14	32.31	13	32.13	12	−	−	−	−	32.09	10
SGMI + NP	34.59	11	37.92	12	45.15	9	60.46	3	32.79	10	45.40	7
SFI + NP	30.29	13	21.53	14	60.86	7	33.83	10	40.21	7	87.61	2
JOEI + NP	33.38	12	95.44	1	29.87	13	47.00	7	24.42	11	7.12	11
NP	10.57	15	2.28	15	1.95	14	2.54	11	5.62	12	6.537	12

Note: The surface under the cumulative ranking curve (SUCRA) was used to estimate the ranking probabilities for different CHIs. A better treatment was indicated by a higher SUCRA value. ADI, Aidi injection; AI, *Astragalus* injection; CSI, Chansu injection; CKSI, Compound kushen injection; DLSI, Delisheng injection; HCSI, Huachansu injection; JOEI, Javanica oil emulsion injection; KAI, Kangai injection; KLTI, Kanglaite injection; NP, vinorelbine plus cisplatin; SFI, Shenfu injection; SMI, Shenma injection; SQFZI, Shenqifuzheng injection; SGMI, Shengmai injection; SUCRA, surface under cumulative the ranking curve; XAPI, Xiaoaiping injection.

#### Performance Status

A total of 63 RCTs contributed to the evidence network for performance status across the 14 types of CHIs. The results indicated that patients receiving 10 types of CHIs: Aidi injection, Compound kushen injection, Huachansu injection, Javanica oil emulsion injection, Kangai injection, Kanglaite injection, Shengmai injection, Shenmai injection, Shenqifuzheng injection, and Xiaoaiping injection, plus NP exhibited considerable improvements in performance status relative to those receiving NP alone. There were significant differences between these groups (Table 3). Compared with Aidi injection plus NP, Compound kushen injection plus NP, Huachansu injection plus NP, Kangai injection plus NP, Kanglaite injection plus NP, Shenfu injection plus NP and Shengmai injection plus NP, Javanica oil emulsion injection plus NP might hold greater potential for improvements in performance status (Table 2). The combination of Javanica oil emulsion injection and NP seemed to be the best intervention for performance status, with a SUCRA value of 95.44%, followed by Xiaoaiping injection plus NP (81.01%) and Aidi injection plus NP (70.37%) (Figure 5B). The SUCRA values of the other CHIs are summarized in Table 3.

#### Leukopenia

There were 78 RCTs with 13 CHIs involved in the NMA concerning leukopenia. As shown in Table 4, Aidi injection, Compound kushen injection, Delisheng injection, Huachansu injection, Javanica oil emulsion injection, Kangai injection, Kanglaite injection, Shenfu injection, Shengmai injection, Shenmai injection and Shenqifuzheng injection were 11 injections combined with NP, which demonstrated a favorable trend for relieving leukopenia compared with NP alone. There were significant differences between these groups. Compared with Javanica oil emulsion injection plus NP, Shenmai injection plus NP might hold greater potential for relieving leukopenia. The results of the SUCRA analysis showed that Shenmai injection plus NP (86.87%) seemed to be the most tolerable option for significantly relieving leukopenia, followed by Shenqifuzheng injection plus NP (61.74%) and Huachansu injection plus NP (61.72%) (Figure 5C). The SUCRA values of each treatment in terms of different outcomes are displayed in Table 3.

**TABLE 4:** Results of the network meta-analysis of leukopenia (upper-right quarter) and hemoglobin reduction (lower left quarter).

ADI + NP	3.22 (2.39.4.39)	1.59 (0.37.6.61)	0.92 (0.52.1.63)	0.90 (0.32.2.48)	0.90 (0.35.2.27)	1.34 (0.78.2.32)	0.93 (0.53.1.68)	1.17 (0.67.1.99)	0.93 (0.49.1.75)	1.13 (0.56.2.27)	0.64 (0.35.1.18)	0.92 (0.53.1.62)	1.31 (0.34.4.95)
**1.78 (1.13,2.79)**	**NP**	0.49 (0.12.1.98)	**0.29 (0.17,0.46)**	**0.28 (0.10,0.73)**	**0.28 (0.11,0.66)**	**0.42 (0.27,0.65)**	**0.29 (0.18,0.47)**	**0.36 (0.22,0.56)**	**0.29 (0.16,0.50)**	**0.35 (0.18,0.66)**	**0.20 (0.12,0.34)**	**0.29 (0.18,0.46)**	0.41 (0.11.1.47)
−	−	**AI + NP**	0.58 (0.13.2.63)	0.56 (0.10.3.17)	0.56 (0.11.3.07)	0.84 (0.20.3.83)	0.58 (0.13.2.69)	0.73 (0.17.3.31)	0.58 (0.13.2.72)	0.71 (0.15.3.42)	0.40 (0.091.1.87)	0.58 (0.13.2.65)	0.82 (0.12.5.69)
0.80 (0.33.1.90)	**0.45 (0.21,0.94)**	−	**CKSI + NP**	0.98 (0.32.2.88)	0.98 (0.36.2.66)	1.45 (0.76.2.87)	1.006 (0.51.2.05)	1.27 (0.65.2.45)	1.007 (0.48.2.12)	1.22 (0.55.2.73)	0.70 (0.34.1.44)	1 (0.51.1.98)	1.42 (0.35.5.67)
**0.081 (0.013,0.37)**	**0.046 (0.0076,0.19)**	−	**0.10 (0.015,0.52)**	**DLSI + NP**	1 (0.27.3.76)	1.49 (0.52.4.49)	1.034 (0.35.3.13)	1.30 (0.44.3.84)	1.033 (0.34.3.23)	1.26 (0.39.4.09)	0.72 (0.24.2.18)	1.027 (0.35.3.096)	1.46 (0.28.7.46)
−	−	−	−	−	**HCSI + NP**	1.49 (0.56.4.04)	1.033 (0.38.2.86)	1.30 (0.48.3.47)	1.032 (0.37.2.93)	1.25 (0.42.3.74)	0.72 (0.26.2.009)	1.026 (0.38.2.78)	1.46 (0.30.6.86)
0.85 (0.42.1.71)	**0.48 (0.28,0.81)**	−	1.055 (0.43.2.69)	**10.48 (2.25,68.98)**	−	**JOEI + NP**	0.69 (0.36.1.35)	0.87 (0.45.1.62)	0.69 (0.34.1.40)	0.84 (0.38.1.81)	**0.48 (0.24,0.96)**	0.69 (0.36.1.31)	0.97 (0.24.3.81)
0.91 (0.35.2.39)	0.51 (0.21.1.20	−	1.14 (0.36.3.62)	**11.16 (2.046,82.62)**	−	1.076 (0.38.2.93)	**KAI + NP**	1.26 (0.62.2.41)	1 (0.47.2.083)	1.21 (0.54.2.69)	0.70 (0.33.1.42)	0.99 (0.5.1.94)	1.41 (0.34.5.56)
−	−	−	−	−	−	−	−	**KLTI + NP**	0.79 (0.39.1.66)	0.96 (0.45.2.13)	0.55 (0.28.1.12)	0.79 (0.42.1.54)	1.12 (0.28.4.49)
1.038 (0.47.2.23)	0.58 (0.30.1.08)	−	1.29 (0.48.3.53)	**12.69 (2.61,84.04)**	−	1.22 (0.53.2.76)	1.12 (0.39.3.32)	−	**SFI + NP**	1.21 (0.52.2.83)	0.69 (0.32.1.51)	1 (0.48.2.073)	1.41 (0.34.5.78)
0.66 (0.22.1.96)	**0.37 (0.14,0.99)**	−	0.82 (0.23.2.92)	**8.30 (1.4,63.03)**	−	0.78 (0.26.2.36)	0.73 (0.20.2.59)	−	0.64 (0.20.2.076)	**SGMI + NP**	0.57 (0.25.1.30)	0.82 (0.37.1.81)	1.16 (0.27.4.89)
0.50 (0.19.1.25)	**0.28 (0.12,0.63)**	−	0.61 (0.20.1.89)	**6.039 (1.12,40.72)**	−	0.58 (0.22.1.52)	0.54 (0.16.1.85)	−	0.47 (0.17.1.37)	0.75 (0.21.2.69)	**SMI + NP**	1.43 (0.71.2.90)	2.022 (0.49.8.25)
0.76 (0.39.1.50)	**0.43 (0.25,0.72)**	−	0.95 (0.39.2.43)	**9.41 (1.98,60.17)**	−	0.89 (0.43.1.89)	0.83 (0.31.2.34)	−	0.73 (0.33.1.68)	1.15 (0.39.3.52)	1.55 (0.57.4.23)	**SQFZI + NP**	1.41 (0.35.5.62)
0.84 (0.24.2.79)	0.47 (0.14.1.43)	−	1.047 (0.26.4.051)	**10.17 (1.62,86.22)**	−	0.98 (0.27.3.47)	0.92 (0.21.3.96)	−	0.81 (0.21.2.96)	1.26 (0.28.5.60)	1.67 (0.41.7.11)	1.10 (0.30.3.76)	**XAPI + NP**

Note: The differences between the compared groups were deemed as significant when the 95% CI of the OR did not contain 1.00, which is marked as bold font. ADI, Aidi injection; AI, *Astragalus* injection; CSI, Chansu injection; CKSI, Compound kushen injection; DLSI, Delisheng injection; HCSI, Huachansu injection; JOEI, Javanica oil emulsion injection; KAI, Kangai injection; KLTI, Kanglaite injection; NP, vinorelbine plus cisplatin; SFI, Shenfu injection; SMI, Shenma injection; SQFZI, Shenqifuzheng injection; SGMI, Shengmai injection; XAPI, Xiaoaiping injection.

#### Hemoglobin Reduction

A total of 27 RCTs with 10 CHIs provided information on hemoglobin reduction in the NMA. The results indicated that Aidi injection, Compound kushen injection, Delisheng injection, Javanica oil emulsion injection, Shengmai injection, Shenmai injection and Shenqifuzheng injection in combination with NP were significantly different from NP alone with respect to lowering the level of hemoglobin (Table 4). The SUCRA results showed that Delisheng injection combined with NP (99.54%) might be a more suitable option than the other types of CHIs for patients with NSCLC experiencing hemoglobin reduction, followed by Shenmai injection plus NP (76.26%) and Shengmai injection plus NP (60.46%) (Figure 5D). The SUCRA values of the other options are shown in Table 3.

#### Thrombocytopenia

Data on thrombocytopenia were available for 36 RCTs involving 11 CHIs. The results showed that the combination of Aidi injection, Huachansu injection, Kangai injection, Kanglaite injection, Shenfu injection, Shenmai injection or Shenqifuzheng injection with NP could significantly improve thrombocytopenia compared with NP alone (Table 5). The SUCRA results illustrated that Huachansu injection plus NP (92.67%), Aidi injection plus NP (76.20%) and Shenmai injection plus NP (74.17%) were regarded as more efficient in relieving thrombocytopenia than the other types of CHIs. (Figure 5E). The SUCRA values of each intervention in terms of different outcomes are shown in Table 3.

**TABLE 5:** Results of the network meta-analysis of thrombocytopenia (upper-right quarter) and gastrointestinal reaction (lower left quarter).

ADI + NP	2.96 (1.97.4.62)	−	1.66 (0.68.3.97)	0.49 (0.13.1.75)	2.11 (0.96.4.77)	1.26 (0.68.2.39)	1.24 (0.65.2.42)	1.64 (0.81.3.25)	1.91 (0.66.5.54)	0.96 (0.36.2.47)	1.33 (0.70.2.56)	1.72 (0.48.6.04)
3.59 (2.64,5.011)	NP	−	0.56 (0.25.1.19)	0.17 (0.045,0.54)	0.71 (0.37.1.39)	0.42 (0.26,0.67)	0.42 (0.25,0.68)	0.55 (0.31,0.94)	0.65 (0.24.1.68)	0.32 (0.14,0.75)	0.45 (0.27,0.73)	0.58 (0.18.1.90)
2.032 (0.50.8.089)	0.57 (0.14.2.16)	AI + NP	−	030 (0.066.1.22)	1.27 (0.46.3.57)	0.76 (0.3.1.89)	0.75 (0.29.1.91)	0.98 (0.37.2.66)	1.17 (0.33.3.92)	0.57 (0.18.1.81)	0.80 (0.33.2.11)	1.037 (0.24.4.34)
0.99 (0.56.1.76)	0.28 (0.17,0.44)	0.49 (0.11.2.072)	CKSI + NP	−	4.31 (1.097,17.85)	2.56 (0.70.10.18)	2.53 (0.69.9.99)	3.34 (0.89.13.6)	3.88 (0.86.18.58)	1.93 (0.45.9.15)	2.71 (0.75.10.23)	3.51 (0.63.21.06)
0.50 (0.17.1.38)	0.14 (0.049,0.36)	0.24 (0.044.1.31)	0.51 (0.16.1.47)	HCSI + NP	−	0.59 (0.27.1.32)	0.59 (0.25.1.34)	0.776 (0.33.1.76)	0.90 (0.28.2.85)	0.45 (0.15.1.32)	0.63 (0.28.1.41)	0.81 (0.20.3.23)
3.61 (1.88,7.22)	1.007 (0.56.1.83)	1.78 (0.42.7.83)	3.64 (1.72,7.89)	7.21 (2.38,24.34)	JOEI + NP	−	0.99 (0.49.1.92)	1.3 (0.63.2.62)	1.53 (0.51.4.53)	0.76 (0.28.1.99)	1.069 (0.54.2.036)	1.37 (0.37.4.99)
1.72 (1.028,2.92)	0.48 (0.31,0.73)	0.85 (0.21.3.54)	1.73 (0.92.3.31)	3.44 (1.22,10.63)	0.48 (0.23,0.97)	KAI + NP	−	1.311 (0.63.2.75)	1.54 (0.51.4.68)	0.76 (0.28.2.073)	1.072 (0.54.2.18)	1.38 (0.37.5.13)
1.74 (1.028,2.99)	0.49 (0.31,0.74)	0.86 (0.21.3.59)	1.76 (0.92.3.37)	3.48 (1.24,10.74)	0.48 (0.23,0.99)	1.012 (0.55.1.85)	KLTI + NP	−	1.18 (0.38.3.48)	0.58 (0.21.1.63)	0.82 (0.39.1.73)	1.057 (0.28.3.97)
0.65 (0.30.1.37)	0.18 (0.089,0.36)	0.32 (0.070.1.48)	0.66 (0.28.1.5)	1.30 (0.40.4.55)	0.18 (0.071,0.43)	0.38 (0.17,0.83)	0.37 (0.16,0.83)	SFI + NP	−	0.50 (0.13.1.85)	0.69 (0.24.2.065)	0.90 (0.18.4.31)
1.43 (0.65.3.17)	0.40 (0.19,0.82)	0.70 (0.15.3.30)	1.44 (0.61.3.46)	2.86 (0.84.10.22)	0.40 (0.16,1.00)	0.83 (0.36.1.91)	0.82 (0.35.1.91)	2.19 (0.82.6.089)	SGMI + NP	−	1.40 (0.54.3.87)	1.83 (0.42.7.79)
1.075 (0.53.2.22)	0.30 (0.16,0.57)	0.53 (0.12.2.37)	1.089 (0.49.2.41)	2.16 (0.68.7.27)	0.3 (0.12,0.70)	0.63 (0.29.1.35)	0.62 (0.29.1.36)	1.66 (0.66.4.35)	0.76 (0.29.1.97)	SMI + NP	−	1.30 (0.35.4.70)
1.045 (0.60.1.81)	0.29 (0.18,0.45)	0.51 (0.12.2.15)	1.055 (0.55.2.04)	2.072 (0.73.6.47)	0.29 (0.14,0.59)	0.61 (0.33.1.11)	0.60 (0.32.1.11)	1.60 (0.71.3.68)	0.73 (0.31.1.74)	0.97 (0.45.2.11)	SQFZI + NP	−
−	−	−	−	−	−	−	−	−	−	−	−	XAPI + NP

Note: The differences between the compared groups were deemed as significant when the 95% CI of the OR did not contain 1.00, which is marked as bold font. ADI, Aidi injection; AI, *Astragalus* injection; CSI, Chansu injection; CKSI, Compound kushen injection; DLSI, Delisheng injection; HCSI, Huachansu injection; JOEI, Javanica oil emulsion injection; KAI, Kangai injection; KLTI, Kanglaite injection; NP, vinorelbine plus cisplatin; SFI, Shenfu injection; SMI, Shenmai injection; SQFZI, Shenqifuzheng injection; SGMI, Shengmai injection; XAPI, Xiaoaiping injection.

#### Gastrointestinal Reactions

A total of 62 RCTs with 11 CHIs were included in the NMA focused on gastrointestinal reactions. Table 5 presents evidence that the combination of Aidi injection, Compound kushen injection, Huachansu injection, Kangai injection, Kanglaite injection, Shenfu injection, Shengmai injection, Shenmai injection or Shenqifuzheng injection with NP can reduce the risk of gastrointestinal reactions compared with NP alone. The superiority of Huachansu injection plus NP (92.52%), Shenfu injection plus NP (87.61%) and Compound kushen injection plus NP (68.01%) over other types of CHIs in relieving gastrointestinal reactions was further confirmed via SUCRA analysis (Figure 5F). The SUCRA values of the other CHIs are summarized in Table 3.

### Cluster Analysis

**FIGURE 6:**
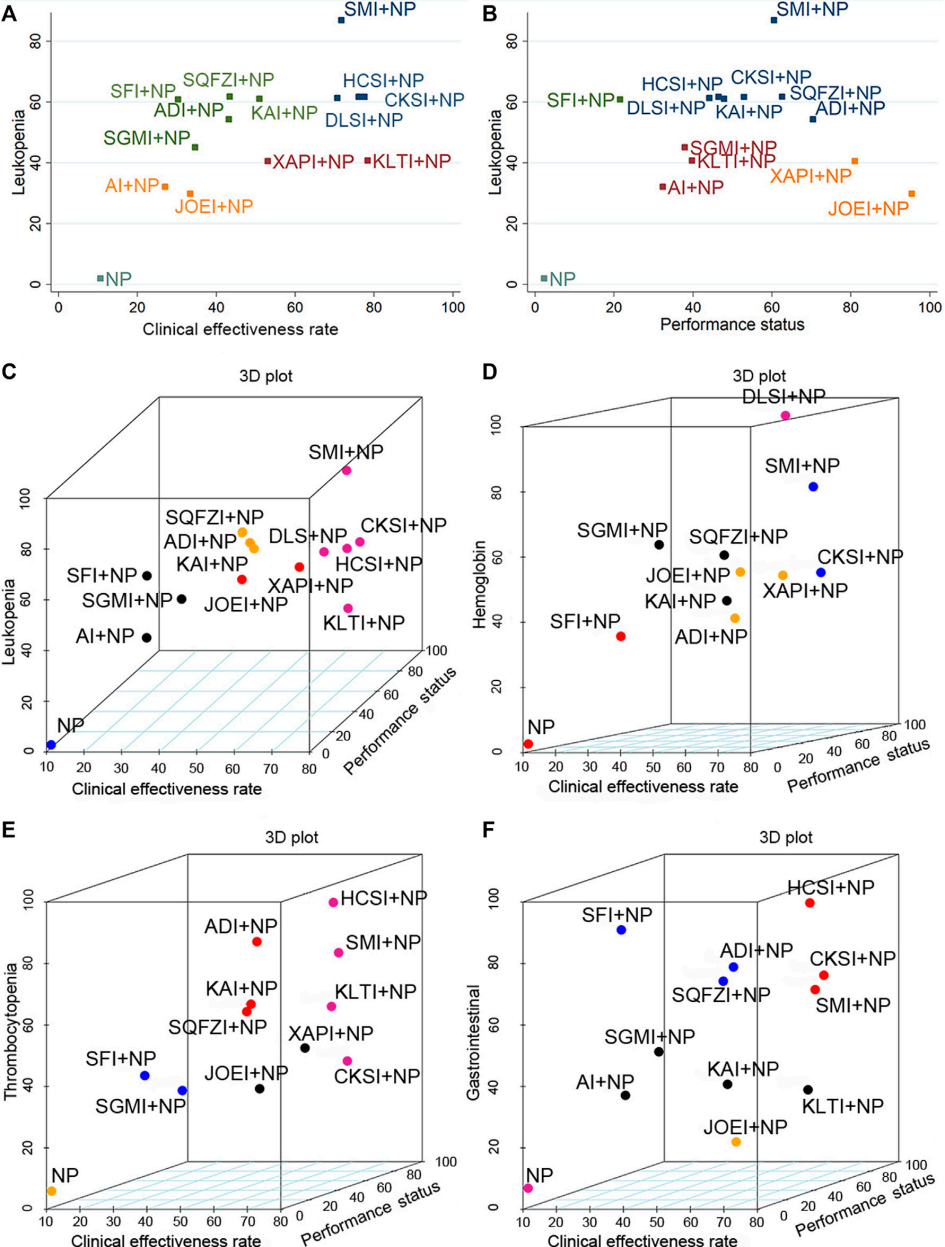
Plots of the cluster analyses for all types of outcomes. **(A)** Clinical effectiveness rate (*x*-axis) and leukopenia (*y*-axis); **(B)** Performance status (*x*-axis) and leukopenia (*y*-axis); **(C)** Clinical effectiveness rate (*x*-axis), performance status (*y*-axis), and leukopenia (*z*-axis); **(D)** Clinical effectiveness rate (*x*-axis), performance status (*y*-axis), and hemoglobin (*z*-axis); **(E)** Clinical effectiveness rate (*x*-axis), performance status (*y*-axis), and thrombocytopenia (*z*-axis); **(F)** Clinical effectiveness rate (*x*-axis), performance status (*y*-axis), and gastrointestinal reactions (*z*-axis). Note: Interventions with the same color belong to the same cluster, and interventions located in the upper-right corner indicate optimal therapy for two different outcomes. NP, vinorelbine and cisplatin; ADI, Aidi injection; AI, *Astragalus* injection; CSI, Chansu injection; CKSI, Compound kushen injection; DLSI, Delisheng injection; HCSI, Huachansu injection; JOEI, Javanica oil emulsion injection; KAI, Kangai injection; KLTI, Kanglaite injection; SFI, Shenfu injection; SMI, Shenmai injection; SQFZI, Shenqifuzheng injection; SGMI, Shengmai injection; XAPI, Xiaoaiping injection.

The cluster analysis was conducted based on the SUCRA values to estimate the safest and most effective treatments. Concerning the efficacy outcomes, the results of the cluster analysis (Figure 6A B) illustrated that among the interventions, Shenmai injection plus NP was superior in improving the clinical effectiveness rate and the performance status and relieving leukopenia. In addition, Kanglaite injection plus NP exhibited a better impact on the clinical effectiveness rate, and Javanica oil emulsion injection plus NP was associated with a preferable response in performance status.

In the multidimensional cluster analysis of the three outcomes of the clinical effectiveness rate, the performance status, and alleviation of leukopenia, NP ranked the worst in relative ranking, and Shenmai injection combined with NP was the most noteworthy (Figure 6C). When it comes to the cluster analysis of interventions that reported the clinical effectiveness rate, the performance status, and alleviation of hemoglobin reduction, NP performed the worst in the comprehensive ranking, and Delisheng injection combined with NP chemotherapy may be the best intervention (Figure 6D). Among the interventions that simultaneously reported the clinical effectiveness rate, the performance status, and relieving thrombocytopenia, the combination of Huachansu injection and NP performed best in the cluster analysis (Figure 6E). And it showed that, Huachansu injection combined with NP were dominant in the comprehensive ranking of the clinical effectiveness rate, the performance status, and alleviation of gastrointestinal reactions (Figure 6F).

### Publication Bias

**FIGURE 7:**
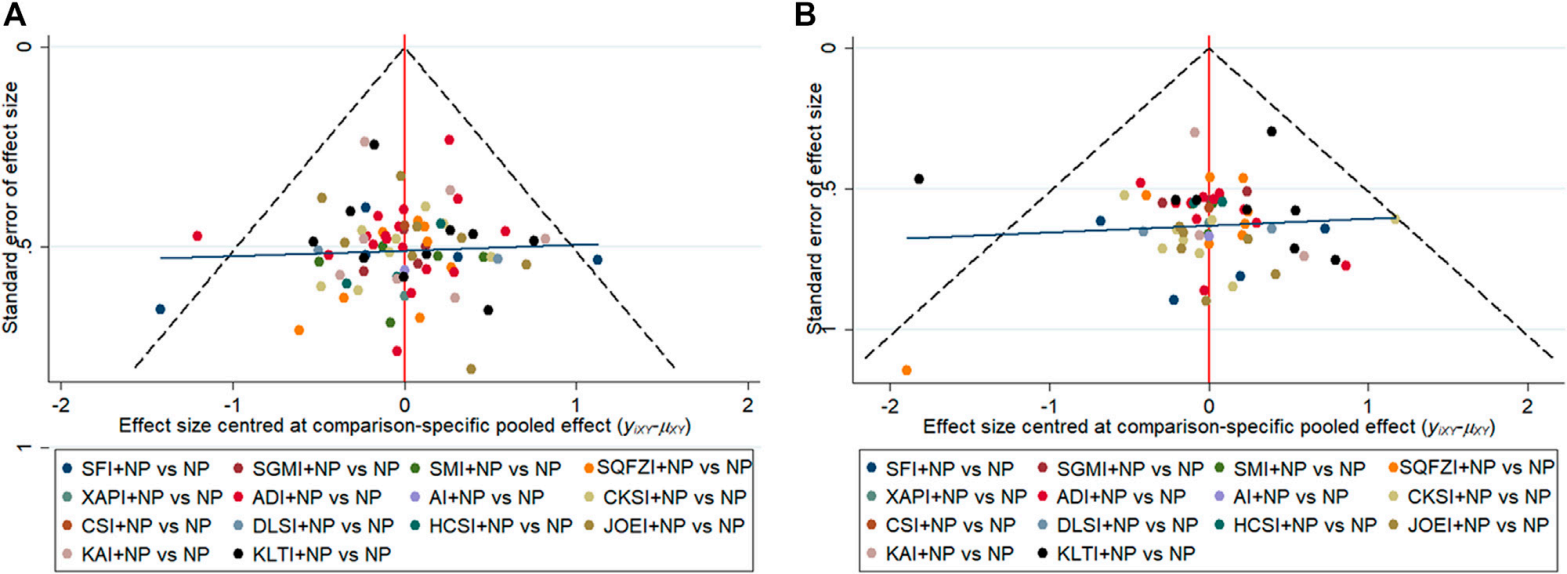
Comparison-adjusted funnel plot for outcomes. **(A)** Clinical effectiveness rate; **(B)** Performance status. Note: NP, vinorelbine and cisplatin; ADI, Aidi injection; AI, *Astragalus* injection; CSI, Chansu injection; CKSI, Compound kushen injection; DLSI, Delisheng injection; HCSI, Huachansu injection; JOEI, Javanica oil emulsion injection; KAI, Kangai injection; KLTI, Kanglaite injection; SFI, Shenfu injection; SMI, Shenma injection; SQFZI, Shenqifuzheng injection; SGMI, Shengmai injection; XAPI, Xiaoaiping injection.

Publication bias was measured by comparison-adjusted funnel plots for efficacy outcomes in this NMA. Visual inspections of the clinical effectiveness rate and performance status revealed that the included RCTs were distributed relatively symmetrically and based on the zero line, and the angle between the adjusted auxiliary line and the zero line was small. Therefore, there may be minor publication bias (Figure 7).

## Discussion

Lung cancer, one of the most malignant tumors, seriously threatens human health (Sun, 2004). As many studies have shown, the combination of CHIs and NP is widely adopted in China and has achieved the desired efficacy (Hofseth and Wargovich, 2007; Konkimalla and Efferth, 2008; Qi et al., 2010). This NMA evaluated 14 types of CHIs combined with NP in the treatment of NSCLC and indicated that all eligible CHIs combined with NP have a positive effect on NSCLC patients. According to the results of the cluster analysis and the ORs, all eligible CHIs combined with NP were associated with a more beneficial effect than NP alone. Moreover, Shenmai injection combined with NP was associated with a preferable response in improving the clinical effectiveness rate and performance status and relieving leukopenia. Hence, the efficacy of Shenmai injection combined with NP should be considered for patients with NSCLC. Moreover, Kanglaite injection plus NP might be a suitable option regarding the clinical effectiveness rate, and Javanica oil emulsion injection plus NP was considered more efficient regarding the performance status. However, clinicians should choose different treatments adhering to the specific requirements of the patients.

Shenmai injection is derived from a famous traditional Chinese herbal prescription and has been used as an injection since 1995, in which the primary pharmacological activity constituents are ginsenosides and Ophiopogon (Xia et al., 2008; Lu et al., 2014; Zhou et al., 2018). Several pharmacological studies have shown that Shenmai injection has immunomodulatory effects on tissue damage by inhibiting the release of the inflammatory products of macrophages, such as tumor necrosis factor alpha (TNF-α) and nitric oxide (NO). It also confers protective effects by expanding coronary arteries, which can increase blood supply throughout the body and improve capillary blood circulation (Bai et al., 2011). Javanica oil emulsion injection combined with NP is estimated as the best intervention in improving the performance status. Pharmacological studies have shown that it has good anti-tumor and anti-malarial effects, which may be due to its main active ingredients including fatty acids, such as oleic acid, palmitic acid, and arachidonic acid (Sun et al., 1981; Li et al., 2004). Their anti-tumor mechanisim may be that they can inhibit the growth of tumor cells, block their cell cycle, and specifically destroy the membrane and mitochondria of cancer cells. And it is worth noting that they have the potential to reverse drug resistance (Ding and Suo, 2006; Jiang et al., 2011).

Currently, there has been only one previous NMA that evaluated CHIs combined with NP for treating NSCLC, which was published in 2015 and included 167 RCTs involving 4,480 participants (Tian et al., 2015). In contrast, this NMA has the following merits. First, this NMA included comprehensive coverage of the current and latest research findings with the aim of ensuring the objectivity and authenticity of the results. Second, the eligibility criteria were formulated and established strictly in this NMA. Specifically, the patients of the included RCTs were restricted to having stage Ⅲ or Ⅳ NSCLC to reduce the interference of clinical heterogeneity. Finally, in this NMA, hemoglobin reduction and thrombocytopenia were added as outcome indicators for patients, and the cluster analysis was performed based on the SUCRA values to select the superior CHIs in terms of efficacy.

Several unavoidable limitations of the current NMA should be noted. First, this NMA did not estimate the results of long-term endpoint outcomes due to insufficient information, while the survival time or survival rate were regarded as vital in identifying and judging the therapeutic effects of patients with cancer. Second, some of the included RCTs did not perform a dialectical analysis. Systematic reviews should synthesize the data according different types of symptoms and provide clearer guidelines for clinical practice. Third, the methodological quality of the included RCTs was not high. The percentage of RCTs that provided details in regard to the randomization method, allocation concealment and blinding methods and the sample size in each study were relatively small. For this reason, we recommend that RCTs should be registered ahead of time to ensure the transparency of the process and improve the methodological quality. Second, the implementation of RCTs should abide by the latest clinical diagnosis and treatment guidelines. Third, RCTs of patients with cancer should focus on long-term and valuable endpoints. In terms of the above limitations, more rigorous RCTs with high quality are needed to verify the value of CHIs combined with NP for patients with NSCLC.

## Conclusion

In general, the current evidence indicates that CHIs combined with NP might have a more beneficial effect on NSCLC patients than NP alone. Among the 14 interventions, Shenmai injection plus NP, Kanglaite injection plus NP and Javanica oil emulsion injection plus NP were found to be preferable interventions for NSCLC. Owing to dialectical analysis within the study populations and the methodological quality of the included RCTs, more multicenter and better designed RCTs are needed to support the findings in this NMA.

## Abbreviations

95% CI, 95% credible interval; ADRs/ADEs, adverse drug reactions/adverse drug events; CHIs, Chinese herbal injections; CR, complete response; KPS, Karnofsky performance status; NMA, network meta-analysis; NSCLC, non-small cell lung cancer; NP, vinorelbine and cisplatin; OR, odds ratio; PD, progressive disease; PR, partial response; RCTs, randomized controlled trials; SD, stable disease; SUCRA, surface under the cumulative ranking curve.
