# Supplementary material for: Investigation on the Efficiency of Chinese Herbal Injections for Treating Non-small Cell Lung Cancer With Vinorelbine and Cisplatin Based on Multidimensional Bayesian Network Meta-Analysis
Source: Front Pharmacol. 2021 Jan 29;11:631170. doi: 10.3389/fphar.2020.631170 (PMC7941272; doi:10.3389/fphar.2020.631170)
Supplement: Supplementary file 1 [file datasheet1.pdf]

PRISMA 2009 Checklist

Section/Topic	#	Checklist Item	Reported on Page #
**TITLE**			
Title	1	Identify the report as a systematic review, meta-analysis, or both.	1
**ABSTRACT**			
Structured summary	2	Provide a structured summary including, as applicable: background; objectives; data sources; study eligibility criteria, participants, and interventions; study appraisal and synthesis methods; results; limitations; conclusions and implications of key findings; systematic review registration number.	1-2
**INTRODUCTION**			
Rationale	3	Describe the rationale for the review in the context of what is already known.	2
Objectives	4	Provide an explicit statement of questions being addressed with reference to participants, interventions, comparisons, outcomes, and study design (PICOS).	2-3
**METHODS**			
Protocol and registration	5	Indicate if a review protocol exists, if and where it can be accessed (e.g., Web address), and, if available, provide registration information including registration number.	/
Eligibility criteria	6	Specify study characteristics (e.g., PICOS, length of follow-up) and report characteristics (e.g., years considered, language, publication status) used as criteria for eligibility, giving rationale.	3-4
Information sources	7	Describe all information sources (e.g., databases with dates of coverage, contact with study authors to identify additional studies) in the search and date last searched.	3
Search	8	Present full electronic search strategy for at least one database, including any limits used, such that it could be repeated.	3
Study selection	9	State the process for selecting studies (i.e., screening, eligibility, included in systematic review, and, if applicable, included in the meta-analysis).	4-5
Data collection process	10	Describe method of data extraction from reports (e.g., piloted forms, independently, in duplicate) and any processes for obtaining and confirming data from investigators.	4-5
Data items	11	List and define all variables for which data were sought (e.g., PICOS, funding sources) and any assumptions and simplifications made.	4-5
Risk of bias in individual studies	12	Describe methods used for assessing risk of bias of individual studies (including specification of whether this was done at the study or outcome level), and how this information is to be used in any data synthesis.	4-5
Summary measures	13	State the principal summary measures (e.g., risk ratio, difference in means).	5
Synthesis of results	14	Describe the methods of handling data and combining results of studies, if done, including measures of consistency (e.g., I^2) for each meta-analysis.	5

PRISMA 2009 Checklist

Section/Topic	#	Checklist Item	Reported on Page #
Risk of bias across studies	15	Specify any assessment of risk of bias that may affect the cumulative evidence (e.g., publication bias, selective reporting within studies).	5
Additional analyses	16	Describe methods of additional analyses (e.g., sensitivity or subgroup analyses, meta-regression), if done, indicating which were pre-specified.	5
**RESULTS**			
Study selection	17	Give numbers of studies screened, assessed for eligibility, and included in the review, with reasons for exclusions at each stage, ideally with a flow diagram.	6
Study characteristics	18	For each study, present characteristics for which data were extracted (e.g., study size, PICOS, follow-up period) and provide the citations.	6
Risk of bias within studies	19	Present data on risk of bias of each study and, if available, any outcome level assessment (see item 12).	6
Results of individual studies	20	For all outcomes considered (benefits or harms), present, for each study: (a) simple summary data for each intervention group (b) effect estimates and confidence intervals, ideally with a forest plot.	6-8
Synthesis of results	21	Present the main results of the review. If meta-analyses done, include for each, confidence intervals and measures of consistency.	9
Risk of bias across studies	22	Present results of any assessment of risk of bias across studies (see Item 15).	9
Additional analysis	23	Give results of additional analyses, if done (e.g., sensitivity or subgroup analyses, meta-regression [see Item 16]).	8
**DISCUSSION**			
Summary of evidence	24	Summarize the main findings including the strength of evidence for each main outcome; consider their relevance to key groups (e.g., healthcare providers, users, and policy makers).	9
Limitations	25	Discuss limitations at study and outcome level (e.g., risk of bias), and at review-level (e.g., incomplete retrieval of identified research, reporting bias).	9-10
Conclusions	26	Provide a general interpretation of the results in the context of other evidence, and implications for future research.	10
**FUNDING**			
Funding	27	Describe sources of funding for the systematic review and other support (e.g., supply of data); role of funders for the systematic review.	10

From: Moher D, Liberati A, Tetzlaff J, Altman DG, The PRISMA Group (2009). Preferred Reporting Items for Systematic Reviews and Meta-Analyses: The PRISMA Statement. PLoS Med 6(6): e1000097. doi:10.1371/journal.pmed1000097

For more information, visit: **www.prisma-statement.org**.

The search strategy used in the English databases

1. Search strategy of Pubmed

#1 Non-Small-Cell Lung Carcinomas [MeSH Terms]
#2 Non-Small-Cell Lung Carcinoma [Title/Abstract]
#3 Nonsmall Cell Lung Cancer [Title/Abstract]
#4 Non Small Cell Lung Carcinoma [Title/Abstract]
#5 Non-Small Cell Lung Carcinoma [Title/Abstract]
#6 Non-Small Cell Lung Cancer [Title/Abstract]
#7 #1 OR #2 OR #3 OR #4 OR #5 OR #6
#8 Shenmai[Title/Abstract]
#9 Delisheng[Title/Abstract]
#10 Astragalus[Title/Abstract]
#11 Huangqi[Title/Abstract]
#12 Shengmai[Title/Abstract]
#13 Chansu[Title/Abstract]
#14 Toad venom[Title/Abstract]
#15 Huachansu[Title/Abstract]
#16 Cinobufacini[Title/Abstract]
#17 Shenfu[Title/Abstract]
#18 Yadanziyouru[Title/Abstract]
#19 Javanica oil emulsion[Title/Abstract]
#20 Xiaoaiping[Title/Abstract]
#21 Marsdenia Tenacissima[Title/Abstract]
#22 Kangai[Title/Abstract]
#23 Aidi[Title/Abstract]
#24 Shenqifuzheng[Title/Abstract]
#25 Kanglaite[Title/Abstract]
#26 Compound matrine[Title/Abstract]
#27 Fufangkushen[Title/Abstract]
#28 Compound Kushen[Title/Abstract]
#29 #8 OR #9 OR #10 OR #11 OR #12 OR #13 OR #14 OR #15 OR #16 OR #17 OR #18 OR #19 OR #20 OR #21 OR #22 OR #23 OR #24 OR #25 OR #26 OR #27 OR #28
#30 randomized controlled trial[Publication Type]
#31 controlled clinical trial[Publication Type]
#32 randomized[Title/Abstract]
#33 placebo[Title/Abstract]
#34 randomly[Title/Abstract]
#35 trial[Title/Abstract]
#36 groups[Title/Abstract]
#37 "drug therapy" [Subheading]
#38 #30 OR #31 OR #32 OR #33 OR # 34 #36 OR #37 OR #38
#39 #7 AND #29 AND #38

2. Search strategy of Embase
#1 random*
#2 placebo*
#3 doubl* adj blind*
#4 singl* adj blind*
#5 assign*
#6 allocat*
#7 “double-blind procedure”/exp
#8 ”randomized controlled trial”/exp
#9”single-blind procedure”/exp
#10 #1 or #2 or #3 or #4 or #5 or #6 or #7 or #8 or #9
#11 " Non-Small-Cell Lung Carcinomas '/exp
#12 Non-Small-Cell Lung Carcinoma
#13 Nonsmall Cell Lung Cancer
#14 Non Small Cell Lung Carcinoma
#15 Non-Small Cell Lung Carcinoma
#16 Non-Small Cell Lung Cancer
#17 #11 OR #12 OR #13 OR #14 OR #15 OR #16
#18 shenmai
#19 delisheng
#20 huangqi
#21 astragalus
#22 shengmai
#23 chansu
#24 toad AND venom
#25 huachansu
#26 cinobufacini
#27 shenfu
#28 yadanziyouru
#29 javanica AND oil AND emulsion
#30 xiaoaiping
#31 marsdenia AND tenacissima
#32 kangai
#33 aidi
#34 shenqifuzheng
#35 kanglaite
#36 compound AND matrine
#37 compound AND kushen
#38 fufangkushen
#39 #18 OR #19 OR #20 OR #21 OR #22 OR #23 OR #24 OR #25 OR #26 OR #27 OR #28 OR #29 OR #30 OR #31 OR #32 OR #33 OR #34 OR #35 OR #36 OR #37 OR #38
#46 #10 AND #17 AND #39

3. Search strategy of Cochrane Library

#1 random*
#2 shenmai: ti,ab,kw
#3 delisheng: ti,ab,kw
#4 huangqi: ti,ab,kw
#5 astragalus: ti,ab,kw
#6 shengmai: ti,ab,kw
#7 chansu: ti,ab,kw
#8 toad AND venom: ti,ab,kw
#9 huachansu: ti,ab,kw
#10 cinobufacini: ti,ab,kw
#11 shenfu: ti,ab,kw
#12 yadanziyouru: ti,ab,kw
#13 javanica AND oil AND emulsion : ti,ab,kw
#14 xiaoaiping: ti,ab,kw
#15 marsdenia AND tenacissima: ti,ab,kw
#16 kangai: ti,ab,kw
#17 aidi: ti,ab,kw
#18 shenqifuzheng: ti,ab,kw
#19 kanglaite: ti,ab,kw
#20 compound AND matrine: ti,ab,kw
#21 compound AND kushen: ti,ab,kw
#22 fufangkushen: ti,ab,kw
#23 #2 or #3 or #4 or #5 or #6 or #7 or #8 or #9 #10 #11 OR #12 OR #13 OR #14 OR #15 OR #16 #17 #18 OR #19 OR #20 OR #21 OR #22
#24 Non-Small-Cell Lung Carcinomas: MeSH descriptor
#25 #1 AND #23 AND #24

Information about the included injections

Name of injection	Source	Species /Raw materials	Botanical plant names	Component ingredients to be measured	Therapeutic claims in TCM	Indication	Adverse drug reactions	Quality control reported? (Y/N)	Chemical analysis reported? (Y/N)
Aidi injection	Guizhou Yibai Pharmaceutical Co., Ltd.	*Mylabris* 1.5g (animal drug), *Ginseng Radix Et Rhizoma* 50g, *Astmgali Radix* 100g, *Acanthopanacis Senticosi Radix Et Rhizoma* Seu Caulis 150g	*Panax ginseng C.A.Mey.;* *Astragalus mongholicus Bunge.*; *Eleutherococcus senticosus (Rupr. & Maxim.) Maxim.*	Ginsenoside Re, ＞2mg/10mL; Cantharidin, 0.008-0.030mg/10mL	Clearing heat and detoxifying, dissipating blood stasis and removing knots	Primary liver cancer, lung cancer, rectal cancer, malignant lymphoma, gynecological malignant tumors, etc.	Patients occasionally have flushing, urticaria, fever, and very few patients have palpitations, chest tightness, nausea and other reactions.	Y-National Pharmaceutical Standard Z52020236; Standard number: WS_3-B-3809-99-2002	N
Astragalus injection	Harbin Zhenbao Pharmaceutical Co., Ltd.; Shanghai Fuda Pharmaceutical Co., Ltd.;	*Astmgali Radix* 20g	*Astragalus mongholicus Bunge.*	Astragaloside IV, ＞0.08mg/mL	Immunomodulator	Adjuvant therapy for malignant tumors	Allergic reactions; dyspnea, asthma, chest tightness; phlebitis, atrial	Y-Standard number: WS_3-B-3335-98	N

Chengdu DIAO Jiuhong Pharmaceutical Factory; Zhengdaqingchunbao Pharmaceutical Co., Ltd.; Shanghai Hefeng Pharmaceutical Co., Ltd.; Dali Pharmaceutical Co., Ltd.; WuxiJiMinKeXin ShanHePharmaceutical Co. Ltd.; Shineway Pharmaceutical Group Ltd.; Shanghai Xinya Pharmaceutical Gaoyou Co., Ltd.; Shanxi Jinxin Shuanghe Pharmaceutical	fibrillation; liver damage, vomiting, diarrhea; headache, kidney damage; rare hemolytic anemia

	Co., Ltd.; Heilongjiangzbd Pharmaceutical Co.,Ltd.; Zhejiang Jiuxu Pharmaceutical Co., Ltd.; HARBIN SANCTITY BIOLOGICAL PHARMACEUTICAL CO.,LTD.								
Chansu injection	Jiangsu Pujin Pharmaceutical Co., Ltd.; Anhui Koyo Pharmaceutical Co.,Ltd.	*Bufonis Venenum* 2g (animal drug)	-	Serotonin ,＞18.0 μg/mL	Clearing heat and detoxifying	Acute and chronic suppurative infections; can also be used as an adjuvant antineoplastic drug	Pain, allergic reactions, poor intravenous drip	Y-National Pharmaceutical Standard Z32020694, Z34020603; Standard number: WS_3-B-3354-98	N
Compound kushen injection	Shanxi Zhendong Pharmaceutical Co., Ltd.	*Radix Sophorae Flavescentis* 1400g, Baituling	*Sophora flavescens Aiton*; *Smilax china L.*	Matrine, ＞ 18mg/mL	Clearing heat and dampness , cooling blood and	Cancer pain and bleeding	No obvious systemic side effects, mild irritation when used locally,	Y-National Pharmaceutical Standard Z14021231, Standard	N

		600g			detoxifying, dispersing knots and relieving pain		but good absorption	number: WS_3-B-2752-97	
Delishen g injection	Beijing Zhengbang Pharmaceutical Co., Ltd.	*Red Ginseng*, *Astmgali Radix*, *Bufonis Venenum* (animal drug), *Mylabris* (animal drug)	*Panax ginseng C.A.Mey.; Astragalus mongholicus Bunge.*	Total solids, ＞20mg/mL; Anhydrous glucose, ＞3.5mg/mL; Total amount of bufonis toxin and bufonisin, 12-18μg/mL; Cantharidin, 14-18 μg/mL	Benefiting Qi, promoting healthiness, eliminating symptoms and resolving knots	Syndrome of Qi Deficiency and Stasis in Middle and Late Stage Primary Hepatocellular Carcinoma is characterized by right costal and abdominal mass, pain, abdominal distension and lack of	Frequent urination, urgency, etc., urinary system irritation symptoms, occasionally hematuria and proteinuria can be seen; liver and kidney damage may occur, occasionally nausea and vomiting, abdominal distension.	Y-National Pharmaceutical Standard Z20010135, National Drug Standard WS_3-134(Z-019)-2006(Z)	N

						food, fatigue and fatigue.			
Huachansu injection	Anhui Huarun Jinchan Pharmaceutical Co., Ltd.	Toad skin (animal drug)	-	Serotonin, ＞0.50% (mg/ml)	Detoxification, detumescence and pain relief	Middle and late stage tumors, chronic hepatitis B, etc.	Vascular stimulation; neutropenia; sinus bradycardia or tachycardia, chest tightness, palpitations, blood pressure changes; allergies, anaphylactic shock	Y-National Pharmaceutical Standard Z34020274, National executive standard WS_3-B-3045-98	N
Kangai injection	Changbaishan Pharmaceutical Co., Ltd.	*Astmgali Radix* 300g, *Ginseng Radix Et Rhizoma* 100g, Matrine 10g	*Astragalus mongholicus Bunge.; Panax ginseng C.A.Mey.*	Total amount of ginsenoside Rgl and Re, ＞ 0.1mg/mL	Invigorating Qi, strengthening immune function of the	Primary liver cancer, lung cancer, rectal cancer,	Chills, fever, allergic-like reactions; rash, itching, flushing; nausea, vomiting,	Y-National Pharmaceutical Standard Z20026868, Standard number: Ws-11222	N

		(Chemical medicine)		; Oxymatrine, 9.0-11.0mg/mL	body	malignant lymphoma and gynecological malignant tumors; leukopenia and hypoxia caused by various causes; chronic hepatitis B	abdominal pain; chest tightness, palpitations, dizziness, headache, difficulty breathing, etc.	(ZD-1222)-2002	
Kanglaite injection	Zhejiang Kanglaite Pharmaceutical Co., Ltd.	Coix seed oil for injection 100g, Soy lecithin for injection, Glycerin for injection, Water for Injection	*Coix lacryma-jobi L.*	Glycerol trioleate, ＞12.0mg/g	Reinforcing Qi, nourishing Yin, eliminating symptoms and resolving knots	Primary non-small cell lung cancer and primary liver cancer with deficiency of both qi and Yin and	Occasionally, fat allergies, such as increased body temperature, mild nausea, and chills, most of the symptoms can disappear naturally after 3	Y-National Pharmaceutical Standard Z10970091, National Food and Drug Administration National Drug Standard	N

dampness due to spleen deficiency should not be operated on; combined with radiotherapy and chemotherapy, it has certain enhancement operation; it has certain anti-cachexia and analgesic effect for patients with advanced cancer.	to 5 days of use. Occasional mild phlebitis	WS3-301 (Z-038) -2006 (Z) -2013

Shenfu injection	China Resources Sanjiu (Ya'an) Medical & Pharmaceutical CO., LTD.; Ya'an Sanjiu Pharmaceutical Co., Ltd.	*Red Ginseng*, *Aconiti Lateralis Radix Praeparata*	*Panax ginseng C.A.Mey.; Aconitum carmichaeli Debeaux*	Aconitine, ＜ 0.1mg/mL ; Ginsenoside Rb1, ＞ 0.5mg/mL	Reviving Yang to prevent collapse and Reinforcing Qi and Restoring Yang	Syndrome of syncope of Yang Qi burst; palpitation, panting, cough, stomachache, diarrhea, arthralgia caused by Yang deficiency (Qi deficiency), etc.	Itching, facial flushing; nausea, vomiting, diarrhea; dizziness, headache, chills, high fever, difficulty breathing	Y-National Pharmaceutical Standard Z51020664, Z20043116, Z51021920, Z20043117; Standard number: WS3-B-3427-98	N
Shenmai injection	Ya'an Sanjiu Pharmaceutical Co., Ltd.; Zhengda Qingchunbao Pharmaceutical Co., Ltd.; Hebei Shenwei Pharmaceutical Co., Ltd.;	*Red Ginseng*, *Radix Ophiopogonis*	*Panax ginseng C.A.Mey.; Ophiopogon japonicus (Thunb.) Ker Gawl.*	Ginsenoside Re, 0.80-2.00 mg/mL; Total amount of ginsenoside Rg1 and Re, ＞	Reinforcing Qi and Restoring Yang、replenishing Yin to promote production of body fluids and	Shock of deficiency of both qi and yin, coronary heart virus myocarditis, chronic cor pulmonale	Allergies; systemic damage; shortness of breath, cough; arrhythmia, tachycardia, increased blood pressure; nausea,	Y-National Pharmaceutical Standard Z51021845, Z33020019, Z13020889, Z51021353, Z53021720, Z51021263, Z20093649;	N

Sichuan Chuanda West China Pharmaceutical Co., Ltd.; Yunnan Gejiu Biopharmaceutical Co., Ltd.; Sichuan Shenghe Pharmaceutical Co., Ltd.; Dali Pharmaceutical Co., Ltd.	0.10mg/mL	generating meridian	and granulocytopenia can improve the immune function of cancer patients, enhance their immune function when combined with chemotherapeutic drugs, and reduce the toxic and side effects caused by chemotherapeutic drugs.	vomiting; dizziness, headache; rash, itching, phlebitis, lower back pain, etc.	National Food and Drug Administration National Drug Standard WS3-B-3428-98-2010Z

Shenqifuzheng injection	Livzon Group Limin Pharmaceutical Factory	*Codonopsis Radix*, *Astmgali Radix*	*Codonopsis pilosula (Franch.) Nannf.*; *Astragalus mongholicus Bunge.*	Total solids, ＞13.0mg/mL; Astragaloside IV, 0.12-0.40 mg/mL; Anhydrous glucose, ＞3.5mg/mL	Reinforcing Qi and reinforcing the healthy Qi	Spleen-lung Qi deficiency causes fatigue, lack of Qi and laziness, and dizziness due to spontaneous sweating. Auxiliary treatment for lung cancer and gastric cancer with the above symptoms	Mild bleeding, low fever, stomatitis, drowsiness	Y-National Pharmaceutical Standard Z19990065, Standard Number WS3-387(Z-50)-2003(Z)-2011	N
Shengmai injection	Changshu Leiyunshang Pharmaceutical Co., Ltd.; Shanghai	*Red Ginseng* 100g, *Radix Ophiopogonis* 312g, *Schisandrae*	*Panax ginseng C.A.Mey.; Ophiopogon*	Ginsenoside Rg1, ＞0.08mg/mL;	Reinforcing Qi, nourishing Yin, restoring	Palpitation, shortness of breath, cold limbs, sweating,	Allergic reactions; systemic damage; rash, itching,	Y-National Pharmaceutical Standard Z20044155, Z31020219,	N

Hutchison Pharmaceutical Co., Ltd.; Ya'an Sanjiu Pharmaceutical Co., Ltd.; Jiangsu suzhong pharmaceutical group co. LTD.; Sichuan Chuanda West China Pharmaceutical Co., Ltd.; Wuliangye Group Yibin Pharmaceutical Co., Ltd.; Shanxi Taihang Pharmaceutical Co., Ltd.; Jilin Jian Yisheng Pharmaceutical Co., Ltd.	*Chinensis Fructus* 156g	*japonicus (Thunb.) Ker Gawl.; Schisandra chinensis (Turcz.) Baill.*	Ginsenoside Re, ＞ 0.04mg/mL; Schisandrin A, ＞ 3μg/mL	meridian and securing the prolapse	desperate pulse and myocardial infarction, cardiogenic shock, septic shock according to the above syndromes	sweating; digestive system damage; heart palpitations, increased blood pressure, arrhythmia, decreased blood pressure; damage to the mental and nervous system; respiratory system damage; phlebitis, local pain; Pain, myalgia, abnormal vision	Z51021882, Z20053993, Z51021356, Z51022475, Z14020812, Z22025251; Executive Standard WS3-B-2865-98

Xiaoaiping injection	Tonghua Jinma Pharmaceutical Group Co., Ltd.; Nanjing Shenghe Pharmaceutical Co., Ltd.	*Marsdeniae Tenacissimae Caulis* Extract 200ml	*Marsdenia tenacissima (Roxb.) Moon*	Total solids, 35.0-45.0 mg/mL; Chlorogenic acid, 9.0-13.0mg/mL	Clearing heat and detoxifying, resolving phlegm and softening hard mass	Esophageal cancer, gastric cancer, lung cancer and liver cancer can be treated with adjuvant therapy of radiotherapy and chemotherapy.	Allergic reactions, anaphylactic shock; migratory muscle pain, joint pain; systemic reactions; rash, itching, sweating; digestive system damage; respiratory system damage; chest tightness, palpitations, increased or decreased blood pressure; dizziness, headache; injection Site pain, phlebitis	Y-National Pharmaceutical Standard Z20025869, Z20025868; Standard Number WS-10630-(ZD-0630)-2002	N
Javanica oil	Guangzhou Baiyunshan	*Bruceae Fructus* oil	*Brucea javanica*	Oleic acid,	Anticancer drug	Lung cancer,	No obvious toxic and side	Y-National Pharmaceuti	N

emulsion injection	Mingxing Pharmaceutical Co., Ltd.; Jiangsu Jiuxu Pharmaceutical Co., Ltd.; Pharmaceutical University of LeiYunShang Pharmaceutical Co. Ltd.	100mL	*(L.) Merr.*	9.0-12.0% (g/g)		brain metastasis of lung cancer and digestive tract tumors	effects, and occasional gastrointestinal discomfort such as greasy feeling, nausea and anorexia	cal Standard Z44021325, Z19993152, Z21020639; Standard number: WS3-B-2739-97

Direct comparison between studies and subgroup analysis results

Subgroup-injection	Clinical effectiveness rate				**Performance status**			
	Number of RCT	Inconsistency	RR (95% CI)	*P* value	Number of RCT	Inconsistency	RR (95% CI)	*P* value
Aidi injection + NP vs NP	19	P=0.86	1.20 (1.07, 1.35)	0.001	12	**P=0.04**	1.47 (1.33, 1.63)	＜0.00001
Chansu injection + NP vs NP	1	-	1.23 (0.76, 1.98)	0.40	1	-	1.26 (0.99, 1.60)	0.07
Delisheng injection + NP vs NP	2	P=0.11	1.43 (0.96, 2.13)	0.08	2	P=0.16	1.26 (1.00, 1.57)	0.05
Compound Kushen injection + NP vs NP	8	P=0.97	1.44 (1.19, 1.73)	0.0002	8	**P=0.03**	1.25 (1.13, 1.37)	＜0.00001
Huachansu injection + NP vs NP	3	P=0.84	1.52 (1.05, 2.19)	0.03	2	P=0.71	1.30 (1.06, 1.60)	0.01
Huangqi injection + NP vs NP	1	-	1.01 (0.56, 1.83)	0.97	1	-	1.16 (0.85, 1.58)	0.35
Kangai injection + NP vs NP	7	P=0.59	1.21 (1.04, 1.40)	0.01	3	P=0.59	1.24 (1.11, 1.38)	0.0001
Kanglaite injection + NP vs NP	10	P=0.62	1.44 (1.23, 1.67)	＜0.00001	8	P=0.07	1.28 (1.17, 1.40)	＜0.00001
Shenfu injection + NP vs NP	6	P=0.09	1.10 (0.90, 1.36)	0.36	4	P=0.24	1.07 (0.98, 1.17)	0.13
Shenmai injection + NP vs NP	7	P=0.56	1.33 (1.10, 1.61)	0.003	4	P=0.87	1.76 (1.33, 2.32)	＜0.0001
Shenqifuzheng injection + NP vs NP	8	P=0.99	1.18 (0.98, 1.42)	0.08	8	P=0.55	1.48 (1.29, 1.70)	＜0.00001
Shengmai injection + NP vs NP	4	P=0.96	1.15 (0.84, 1.58)	0.37	3	P=0.93	1.47 (1.12, 1.94)	0.006
Xiaoaiping injection + NP vs NP	1	-	1.33 (0.53, 3.35)	0.54	1	-	1.77 (1.15, 2.73)	0.010
Yadanziyouru injection + NP vs NP	8	P=0.75	1.12 (0.96, 1.30)	0.15	6	P=1.00	1.58 (1.38, 1.80)	＜0.00001

Subgroup-injection	**Leukopenia**				Hemoglobin reduction			
	Number of RCT	Inconsistency	RR (95% CI)	*P* value	Number of RCT	Inconsistency	RR (95% CI)	*P* value
Aidi injection + NP vs NP	18	**P＜0.00001**	0.63 (0.53, 0.75)	＜0.00001	6	P=0.79	0.65 (0.48, 0.88)	0.005
Delisheng injection + NP vs NP	2	P=0.76	0.65 (0.49, 0.87)	0.004	1	-	0.17 (0.06, 0.50)	0.001
Compound Kushen injection + NP vs NP	8	P=035	0.75 (0.68, 0.84)	＜0.00001	2	P=0.68	0.67 (0.47, 096)	0.03
Huachansu injection + NP vs NP	2	P=0.29	0.64 (0.48, 0.87)	0.004	-	-	-	-
Huangqi injection + NP vs NP	1	-	0.81 (0.56, 1.18)	0.27	-	-	-	-
Kangai injection + NP vs NP	7	**P=0.001**	0.71 (0.56, 0.89)	0.003	2	P=0.68	0.87 (0.74, 1.03)	0.10
Kanglaite injection + NP vs NP	7	**P＜0.00001**	0.60 (0.38, 0.93)	0.02	-	-	-	-
Shenfu injection + NP vs NP	5	P=0.34	0.61 (0.49, 0.77)	＜0.0001	3	P=0.78	0.76 (0.55, 1.04)	0.08
Shenmai injection + NP vs NP	7	P=0.54	0.52 (0.42, 0.64)	＜0.00001	2	P=0.21	0.50 (0.32, 0.79)	0.003
Shenqifuzheng injection + NP vs NP	8	P=0.10	0.65 (0.53, 0.79)	＜0.0001	5	P=0.88	0.58 (042, 0.81)	0.001
Shengmai injection + NP vs NP	4	P=0.93	0.67 (0.54, 0.82)	0.0002	2	P=0.60	0.66 (0.45, 0.98)	0.04
Xiaoaiping injection + NP vs NP	1	-	0.67 (0.40, 1.11)	0.12	1	-	067 (0.36, 1.22)	0.19
Yadanziyouru injection + NP vs NP	8	**P=0.0006**	0.73 (0.57, 0.93)	0.010	3	P=0.90	0.67 (0.51, 0.87)	0.003

Subgroup-injection	Thrombocytopenia				**Gastrointestinal reactions**			
	Number of RCT	Inconsistency	RR (95% CI)	*P* value	Number of RCT	Inconsistency	RR (95% CI)	*P* value
Aidi injection + NP vs NP	8	P=0.60	0.50 (0.39, 0.65)	＜0.00001	14	**P=0.0007**	0.62 (0.52, 0.74)	＜0.00001
Compound Kushen injection + NP vs NP	2	P=0.56	0.69 (0.43, 1.11)	0.13	8	P=0.17	0.76 (0.67, 0.85)	＜0.00001
Huachansu injection + NP vs NP	1	-	0.37 (0.18, 0.74)	0.005	2	P=0.010	0.58 (0.30, 1.12)	0.10
Huangqi injection + NP vs NP	-	-	-	-	1	-	0.80 (0.51, 1.26)	0.34
Kangai injection + NP vs NP	3	P=0.82	0.69 (0.58, 0.82)	＜0.0001	7	**P=0.02**	0.74 (0.59, 0.92)	0.008
Kanglaite injection + NP vs NP	4	P=0.24	0.67 (0.55, 0.82)	0.0001	7	P=0.68	0.66 (0.53, 0.81)	＜0.0001
Shenfu injection + NP vs NP	5	P=0.78	0.66 (0.46, 0.96)	0.03	4	P=0.57	0.63 (0.53, 0.75)	＜0.00001
Shenmai injection + NP vs NP	2	P=0.12	0.55 (0.35, 0.87)	0.010	4	P=0.82	0.60 (0.47, 0.77)	＜0.0001
Shenqifuzheng injection + NP vs NP	6	P=0.93	0.63 (0.49, 0.82)	0.0005	8	P=0.17	0.59 (0.48, 0.74)	＜0.00001
Shengmai injection + NP vs NP	2	P=0.61	0.72 (0.36, 1.43)	0.35	3	P=0.98	0.68 (0.52, 0.89)	0.005
Xiaoaiping injection + NP vs NP	1	-	0.73 (0.35, 1.53)	0.40	-	-	-	-
Yadanziyouru injection + NP vs NP	2	P=0.98	0.78 (0.51, 1.20)	0.26	4	P=0.95	1.00 (0.81, 1.24)	0.99

Summary results of meta regression analysis

<table>
<tr><th rowspan="2">covariate</th><th rowspan="2">groups</th><th colspan="2">Performance status</th><th colspan="2">Leukopenia</th><th colspan="2">Gastrointestinal reactions</th></tr>
<tr><th></th><th>P>|t|</th><th></th><th>P>|t|</th><th></th><th>P>|t|</th></tr>
<tr><td rowspan="5">age</td><td>20-80</td><td></td><td>0.216</td><td></td><td>0.848</td><td></td><td>0.339</td></tr>
<tr><td>30-80</td><td>tau^2 = 0.004833</td><td>0.273</td><td>tau^2 = 0</td><td>0.739</td><td>tau^2 = 0</td><td>0.607</td></tr>
<tr><td>40-80</td><td>I-squared_res = 0.00%</td><td>0.915</td><td>I-squared_res = 0.00%</td><td>0.846</td><td>I-squared_res = 26.36%</td><td>0.685</td></tr>
<tr><td>50-80</td><td>Adj R-squared = -8.87%</td><td>Dropped*</td><td>Adj R-squared = 0%</td><td>0.498</td><td>Adj R-squared = 0%</td><td>0.949</td></tr>
<tr><td>60+</td><td></td><td>0.106</td><td></td><td>Dropped*</td><td></td><td>Dropped*</td></tr>
<tr><td rowspan="6">DDPdose (mg/m^2)</td><td>120</td><td></td><td>Dropped*</td><td></td><td>0.046</td><td></td><td>Dropped*</td></tr>
<tr><td>20-30</td><td>tau^2 = 0</td><td>0.633</td><td>tau2 = 0.04557</td><td>0.457</td><td>tau^2 = 0.06928</td><td>0.986</td></tr>
<tr><td>30-40 (include 30 and 40)</td><td>I-squared_res = 0.00%</td><td>0.588</td><td>I-squared_res = 4.32%</td><td>0.173</td><td>I-squared_res = 32.23%</td><td>0.860</td></tr>
<tr><td>40-50</td><td>Adj R-squared = 0%</td><td>0.381</td><td>Adj R-squared = 8.55%</td><td>0.281</td><td>Adj R-squared = -18.79%</td><td>0.233</td></tr>
<tr><td>60-80 (include 80)</td><td></td><td>0.742</td><td></td><td>0.200</td><td></td><td>0.824</td></tr>
<tr><td>80-100</td><td></td><td>Dropped*</td><td></td><td>Dropped*</td><td></td><td>0.446</td></tr>
</table>

Note: *Which is dropped because of collinearity.
